# Oncogenic KSHV-encoded interferon regulatory factor upregulates HMGB2 and CMPK1 expression to promote cell invasion by disrupting a complex lncRNA-OIP5-AS1/miR-218-5p network

**DOI:** 10.1371/journal.ppat.1007578

**Published:** 2019-01-30

**Authors:** Wan Li, Qingxia Wang, Qi Feng, Fei Wang, Qin Yan, Shou-Jiang Gao, Chun Lu

**Affiliations:** 1 State Key Laboratory of Reproductive Medicine, Nanjing Medical University, Nanjing, P. R. China; 2 Key Laboratory of Pathogen Biology of Jiangsu Province, Nanjing Medical University, Nanjing, P. R. China; 3 Department of Microbiology, Nanjing Medical University, Nanjing, P. R. China; 4 Laboratory of Human Virology and Oncology, Shantou University Medical College, Shantou, Guangdong, P. R. China; 5 UPMC Hillman Cancer Center, Department of Microbiology and Molecular Genetics, University of Pittsburgh, Pittsburgh, PA, United States of America; Wistar Institute, UNITED STATES

## Abstract

Kaposi’s sarcoma (KS), a highly disseminated tumor of hyperproliferative spindle endothelial cells, is the most common AIDS-associated malignancy caused by infection of Kaposi’s sarcoma-associated herpesvirus (KSHV). KSHV-encoded viral interferon regulatory factor 1 (vIRF1) is a viral oncogene but its role in KSHV-induced tumor invasiveness and motility remains unknown. Here, we report that vIRF1 promotes endothelial cell migration, invasion and proliferation by down-regulating miR-218-5p to relieve its suppression of downstream targets high mobility group box 2 (HMGB2) and cytidine/uridine monophosphate kinase 1 (CMPK1). Mechanistically, vIRF1 inhibits p53 function to increase the expression of DNA methyltransferase 1 (DNMT1) and DNA methylation of the promoter of pre-miR-218-1, a precursor of miR-218-5p, and increases the expression of a long non-coding RNA OIP5 antisense RNA 1 (lnc-OIP5-AS1), which acts as a competing endogenous RNA (ceRNA) of miR-218-5p to inhibit its function and reduce its stability. Moreover, lnc-OIP5-AS1 increases DNA methylation of the pre-miR-218-1 promoter. Finally, deletion of vIRF1 from the KSHV genome reduces the level of lnc-OIP5-AS1, increases the level of miR-218-5p, and inhibits KSHV-induced invasion. Together, these results define a novel complex lnc-OIP5-AS1/miR-218-5p network hijacked by vIRF1 to promote invasiveness and motility of KSHV-induced tumors.

## Author summary

Kaposi's sarcoma-associated herpesvirus (KSHV) infection caused Kaposi’s sarcoma (KS), a highly disseminated tumor that frequently occurs in patients with AIDS. KSHV-encoded viral interferon regulatory factor 1 (vIRF1) is an oncogenic protein, which has been shown to be vital in KSHV evasion of innate antiviral response and induction of tumorigenesis but its role in KS tumor invasiveness and motility remains unclear. A growing volume of literatures has proposed that lncRNAs could function as tumor suppressors or oncogenes, and numerous lncRNAs might act as competing endogenous RNAs (ceRNAs) that competitively bind to microRNAs (miRNAs), hence exerting influence on posttranscriptional regulation. However, whether cellular lncRNAs are involved in the progression of KS is still unknown. Here, we revealed a previously undefined role of vIRF1 in cell motility and proliferation, and described the cross-regulatory network of cellular lncRNAs and miRNAs involved in the pathogenesis of KS. We found that the crosstalk between miR-218-5p and lnc-OIP5-AS1 contributed to vIRF1-induced cell motility and proliferation via increasing HMGB2 and CMPK1 expression. In summary, this study constitutes an important discovery related to KS pathogenesis, particularly in the invasiveness and motility of KS tumors.

## Introduction

Kaposi’s sarcoma-associated herpesvirus (KSHV), also known as human herpesvirus 8 (HHV-8), is a double-stranded DNA virus, which belongs to γ-herpesvirus. KSHV was initially identified in an AIDS-associated Kaposi’s sarcoma (AIDS-KS) lesion, and has since been strongly linked to Kaposi’s sarcoma (KS), primary effusion lymphoma (PEL), a subset of multicentric Castleman’s disease (MCD), and KSHV-associated inflammatory cytokine syndrome (KICS) [1]. Like other herpesviruses, the life cycle of KSHV consists of two phases, latent and lytic phases, both of which contribute to KSHV-induced pathogenesis, tumorigenesis and angiogenesis [2, 3]. KSHV genome contains over 90 open reading frames [4], some of which are homologous to human genes. To establish a successful persistent infection, KSHV encodes these homologous proteins to regulate cell growth, immune response, inflammatory response and apoptosis, and thus escape the immune antiviral response of host cells [5]. Moreover, these homologous proteins are also in favor of KSHV-induced tumorigenesis. For examples viral interferon regulatory factors (vIRFs) [6], viral interleukin-6 (vIL-6) [7], viral G protein-coupled receptor (vGPCR) [8], viral Bcl-2 (vBcl-2) [9], viral FLICE inhibitory protein (vFLIP) [10] and viral cyclin (vCyclin) [11] have been shown to be pro-oncogenic or promote tumorigenesis.

The cellular IRFs (IRFs 1~9) are a family of cellular transcription proteins that regulate the expression of interferon and interferon-stimulating genes (ISGs) in innate immune response, among which IRF3 and IRF7 play key roles in the induction and secretion of type I interferon [12]. vIRF1 (449 amino acids), as one of the KSHV vIRFs (vIRF1 to vIRF4), is encoded by KSHV ORF-K9, which has 26.6% and 26.2% of protein homology to cellular IRF3 and IRF7, respectively [13]. vIRF1 has been shown to compete with IRF3 to interact with CBP/p300 coactivators by blocking the formation of CBP/p300-IRF3 complexes, thereby inhibiting IRF3-mediated transcription and signal transduction of type I interferon [14]. However, vIRF1 could not block IRF-7-mediated transactivation [14]. In the other hand, vIRF1 represses tumor suppressor gene p53 phosphorylation, leading to an increase of p53 ubiquitination by reducing ATM kinase activity [15]; vIRF1 could also directly bind to p53 and effectively inhibit p53-mediated apoptosis by reducing its acetylation and inhibiting the transcription of p53 activation [16, 17]. In addition, vIRF1 restrains TGF-beta signaling via direct interaction with Smads (Smad3 and Smad4) to disturb Smad3/Smad4 complexes from binding to DNA and suppresses IRF-1-induced CD95/CD95L signaling-mediated apoptosis [18, 19]. As the first identified oncogenic protein encoded by KSHV, vIRF1 has been reported to transform mouse embryonic fibroblasts (NIH3T3) cells [6], however, its role in KSHV-induced tumor invasiveness and motility and its underlying mechanism remains totally unclear.

Less than 2% of the human genome encodes protein-coding genes, while the vast majority of the genome is transcribed as non-coding RNAs [20]. Based on the size, non-coding RNAs could be vaguely divided into three groups: microRNAs (miRNAs), long non-coding RNAs (lncRNAs), and circular RNAs (circRNAs) [21]. Many miRNAs (~22 nucleotides in length) have been well-characterized and shown to repress gene expression by inhibiting the translation or destabilization of mRNA transcript via binding to mRNA sequences [22]. LncRNAs (>200 nucleotides in length) have indispensable roles in diverse biological processes, including chromatin remodeling, X chromosome inactivation, genomic imprinting, nuclear transport, transcription, RNA splicing and translation [23–25]. A growing volume of literatures support the notion that both lncRNAs and miRNAs could function as tumor suppressors or oncogenes involved in the regulation of cell proliferation, metastasis, apoptosis, and invasion [24, 26, 27]. More interestingly, emerging evidence indicates that numerous lncRNAs might act as competing endogenous RNAs (ceRNAs) that competitively bind miRNAs, hence exerting influence on posttranscriptional regulation [28].

Recently, several oncogenic viruses have been shown to encode lncRNAs and are thought to participate in enhancing viral replication, promoting oncogenesis and contributing to pathogenesis [29–31]. KSHV encodes an lncRNA, known as polyadenylated nuclear RNA (PAN RNA). PAN is multifunctional, regulating KSHV replication, viral and host gene expression, and immune responses [32–35]. However, whether cellular lncRNAs are involved in the progression of KS is still unknown.

In the present work, we aimed to elucidate the role of vIRF1 in cell migration, invasion and proliferation. We found that vIRF1 promoted cell migration, invasion and proliferation by epigenetically silencing miR-218-5p and activating lncRNA-OIP5-AS1 transcription. Further, we uncovered that the crosstalk between miR-218-5p and lnc-OIP5-AS1 contributed to vIRF1-induced cell motility and proliferation via increasing HMGB2 and CMPK1 expression. Our novel findings illustrated a critical role of vIRF1 in the invasiveness, motility and development of KS tumor.

## Results

### Exogenous vIRF1 accelerates endothelial cell motility and proliferation

**Fig 1:**
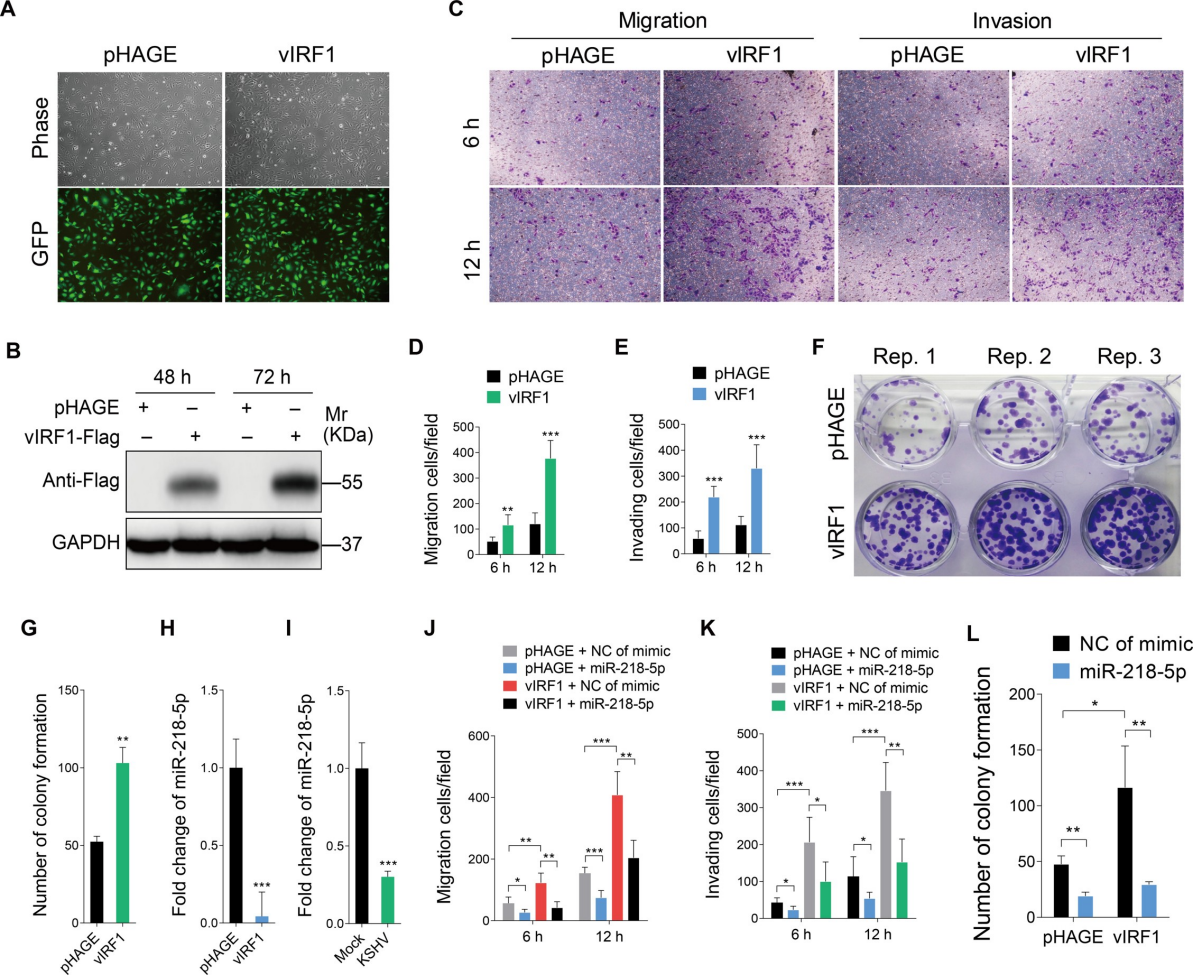
Overexpression of vIRF1 negatively regulates miR-218-5p expression and enhances endothelial cell motility, invasion and proliferation. **(A).** Representational images of HUVECs transduced with vIRF1 (**vIRF1**) or control lentivirus (**pHAGE**). Magnification, ×100. **(B).** Western blotting analysis for vIRF1 protein in HUVECs infected with vIRF1 (**vIRF1**) or control lentivirus (**pHAGE**) for 48 h and 72 h, respectively. The antibody against the Flag-tag was used to detect vIRF1. GAPDH was used as loading control. **(C).** Migration and invasion analysis of HUVECs expressing vIRF1 (**vIRF1**) or control lentivirus (**pHAGE**) at 6 h and 12 h. **(D) and (E).** Quantification of cell migration and invasion described in (**C**), respectively. Results are from three independent experiments, each with quintuple technical replicates, were performed. **(F).** Plate colony formation assay of HUVECs transduced with vIRF1 (**vIRF1**) or control lentivirus (**pHAGE**)**. (G).** Quantification of cell colony formation described in (**F**). Results are from three independent experiments, each with cubic technical replicates, were performed. **(H) and (I).** qPCR showing miR-218-5p expression in vIRF1-transduced and KSHV-infected cells, respectively. **(J).** Migration analysis of vIRF1-infected HUVECs transfected with miR-218-5p mimic for 48 h. **(K).** Invasion analysis of vIRF1-expressing HUVECs treated as in (**J**). **(L).** Colony formation assay of HUVECs treated as in (**J**). The quantified results represent mean ± SD. * *P* < 0.05, ** *P* < 0.01, and *** *P* < 0.001, Student's t-test.

Previous works showed that vIRF1, as a homologue of cellular IRFs, disrupted immune antiviral response of host cells and contributed to KSHV-induced tumorigenesis [5]. However, its role on tumor invasiveness and motility remains unclear. To determine whether vIRF1 had a role in cell motility, we transduced HUVECs with lentiviral vIRF1 at a MOI of 2. vIRF1-transduced HUVECs showed a vIRF1 mRNA expression level similar to that of KSHV-infected HUVECs (S1 Fig, Fig 1A and 1B). We then examined the effect of vIRF1 on cell migration and invasion. In transwell migration and Matrigel invasion assays, overexpression of vIRF1 enhanced cell migration and invasion (Fig 1C, 1D and 1E). In plate colony formation assay, vIRF1 clearly enhanced cell proliferation (Fig 1F and 1G).

### Exogenous vIRF1 expression promotes endothelial cell motility, and proliferation by negatively regulating miR-218-5p expression

To assess the mechanism mediating vIRF1 promotion of cell motility and proliferation, we performed microarray-based miRNA expression profiling and identified a set of miRNAs that were differentially expressed between vIRF1- and pHAGE-transduced HUVECs (GEO accession number GSE119034). As a known tumor suppressor [36], miR-218-5p was significantly down-regulated in vIRF1- transduced cells, and hence was selected for further validation by qRT-PCR. As shown in Fig 1H and 1I, downregulation of miR-218-5p was observed in both vIRF1-transduced and KSHV-infected HUVECs. Then, we sought to determine whether the downregulation of miR-218-5p might contribute to vIRF1 promotion of cell motility, and proliferation. As expected, overexpression of miR-218-5p in vIRF1-transduced HUVECs reversed vIRF1-enhanced cell migration and invasion (S2 Fig, Fig 1J and 1K) as well as cell proliferation (Fig 1L).

### vIRF1 enhances cell motility and proliferation by inhibiting miR-218-5p to increase the expression of its direct targets HMGB2 and CMPK1

**Fig 2:**
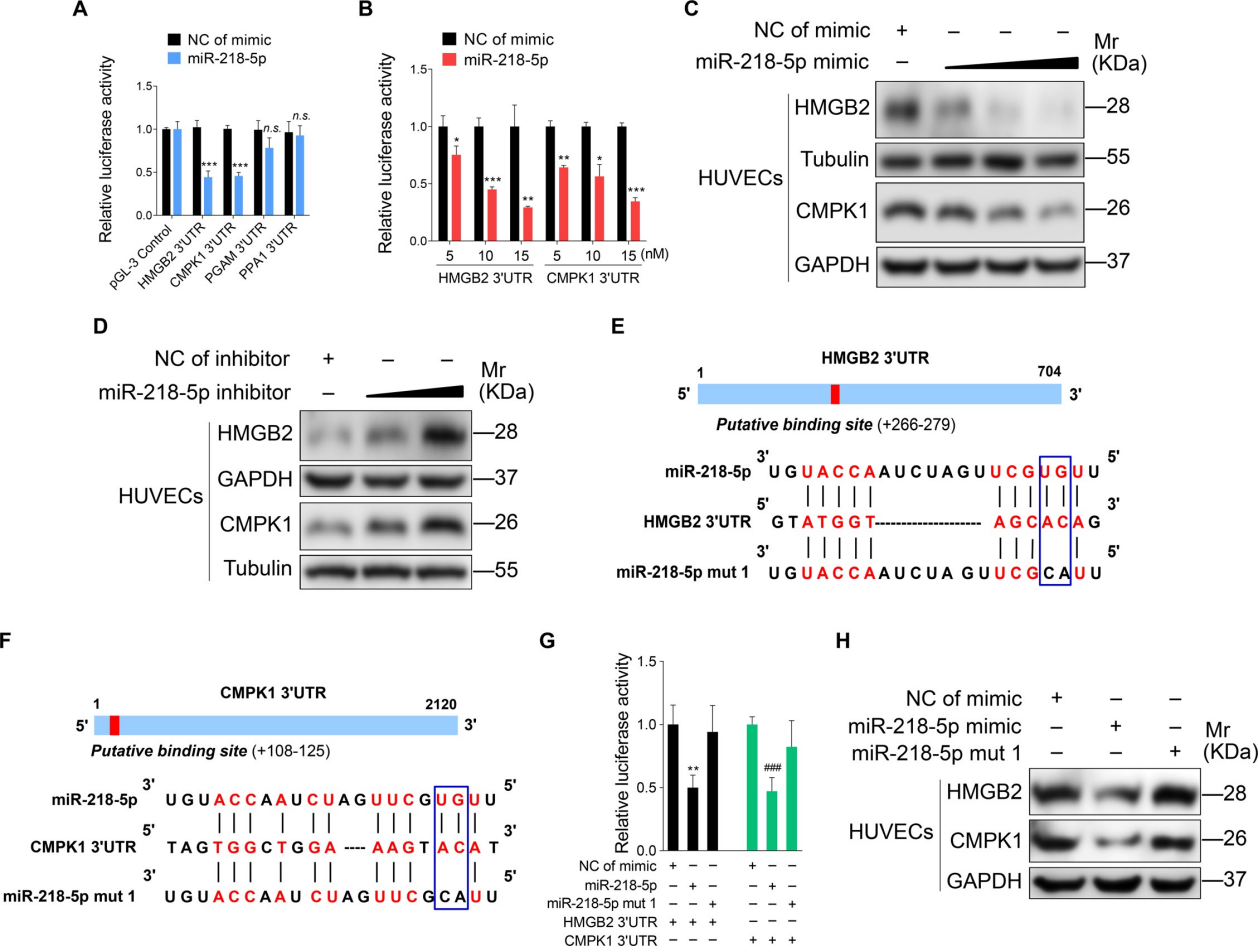
miR-218-5p directly targets HMGB2 and CMPK1 3'UTR. **(A).** Luciferase activity in HEK293T cells cotransfected with miR-218-5p mimic (**miR-218-5p**; 10 nM) or its control (**NC of mimic**; 10 nM) and the reporter constructs for 48 h. **(B).** Luciferase activity in HEK293T cells cotransfected with increasing amounts of miR-218-5p mimic (**miR-218-5p**; 5, 10, and 15 nM) or its control (**NC of mimic**), and the pGL-3-HMGB2 3'UTR reporter or pGL-3-CMPK1 3'UTR reporter for 48 h. **(C).** Western blotting of HMGB2 and CMPK1 expression in HUVECs transfected with increasing amounts of miR-218-5p mimic (10, 20 and 40 nM) or its control for 48 h. **(D).** Western blotting of HMGB2 and CMPK1 expression in HUVECs transfected with a miR-218-5p inhibitor for 48 h. **(E).** Putative binding site of miR-218-5p in the 3'UTR region of HMGB2 and mutagenesis of target site in miR-218-5p. **(F).** Putative binding site of miR-218-5p in the 3'UTR region of CMPK1 and mutagenesis of target site in miR-218-5p. **(G).** Luciferase activity in HEK293T cells cotransfected with miR-218-5p mimic (**miR-218-5p**; 10 nM), miR-218-5p mutant mimic (**miR-218-5p mut 1**; 10 nM) or a negative control (**NC of mimic**; 10 nM), and the HMGB2 3'UTR or CMPK1 3'UTR reporter construct for 48 h. **(H).** Western-blotting of HMGB2 and CMPK1 expression in HUVECs transfected with a negative control mimic (**NC of mimic**; 20 nM), miR-218-5p mimic (**miR-218-5p mimic**; 20 nM) or miR-218-5p mutant mimic (**miR-218-5p mut 1**; 20 nM) for 48 h, respectively. The quantified results represent mean ± SD. Results are from three independent experiments, each with quadruple technical replicates, were performed. * *P* <0.05, ** *P* <0.01, *** *P* < 0.001, and ^###^ *P* < 0.001, Student's t-test. *n*.*s*, not significant.

**Fig 3:**
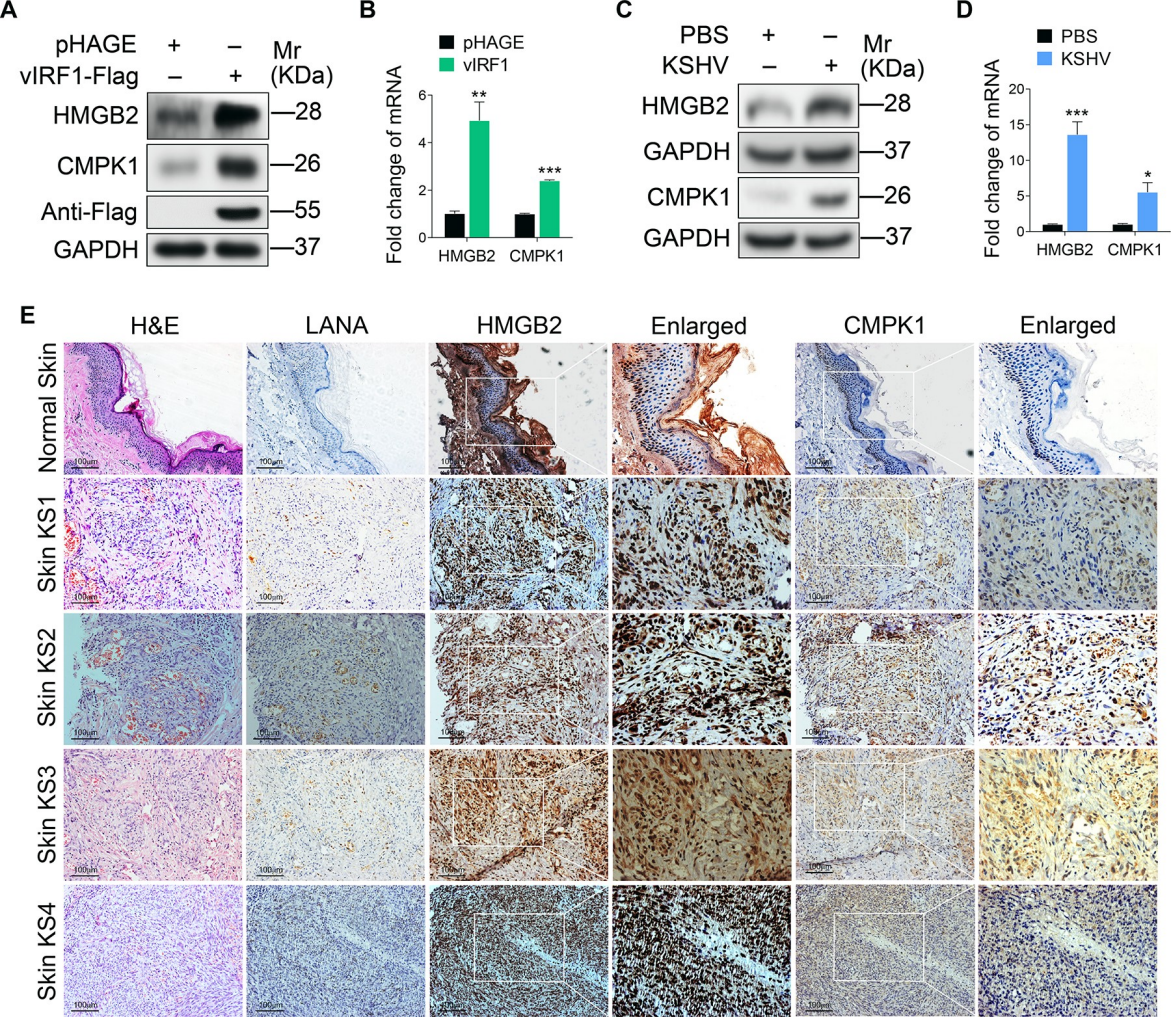
HMGB2 and CMPK1 expression is increased in vIRF1-transduced and KSHV-infected HUVECs, and KS lesion samples. **(A).** Western-blotting of HMGB2 and CMPK1 expression in vIRF1-transduced HUVECs. **(B).** qPCR showing HMGB2 and CMPK1 mRNA transcription in vIRF1-transduced HUVECs. **(C).** Western-blotting of HMGB2 and CMPK1 expression in KSHV-infected HUVECs. **(D).** qPCR showing HMGB2 and CMPK1 mRNA transcription in KSHV-infected HUVECs. **(E).** H&E staining, and immunohistochemical staining of KSHV LANA, HMGB2 and CMPK1 in normal skin, skin KS of patient #1 (**Skin KS1**), skin KS of patient #2 (**Skin KS2**), skin KS of patient #3 (**Skin KS3**) and skin KS patient #4 (**Skin KS4**). Magnification, ×200, ×400. The quantified results represent mean ± SD. Results are from three independent experiments, each with quadruple technical replicates, were performed. * *P* < 0.05, ** *P* < 0.01, and *** *P* < 0.001, Student's t-test.

Next, we conducted mass spectrometry analysis to investigate the direct targets of miR-218-5p. As shown in Table 1, there were a series of proteins that were up-regulated by > 1.5 folds in cells overexpressing vIRF1. Using bioinformatics analysis, we predicted four proteins that might be the potential targets of miR-218-5p, and hence chose them for further luciferase reporter assay. We confirmed that miR-218-5p decreased the 3’UTR reporter activities of both high mobility group box 2 (HMGB2) and cytidine/uridine monophosphate kinase 1 (CMPK1) (Fig 2A), which was further shown in a dose-dependent fashion (Fig 2B). Indeed, overexpression of miR-218-5p suppressed the levels of HMGB2 and CMPK1 proteins in a dose-dependent manner (Fig 2C). Conversely, blocking the miR-218-5p function with a specific inhibitor elevated the expression levels of HMGB2 and CMPK1 proteins in a dose-dependent manner (Fig 2D). To further confirm that miR-218-5p directly targeted HMGB2 and CMPK1, we performed mutagenesis with miR-218-5p (Fig 2E and 2F). The mutant mimic did not have any effect on the 3’UTR reporter activities of HMGB2 and CMPK1 (Fig 2G), and the levels of HMGB2 and CMPK1 proteins (Fig 2H). Moreover, the mRNA and protein levels of HMGB2 and CMPK1 were significantly up-regulated in cells expressing vIRF1 or infected by KSHV (Fig 3A–3D). In IHC staining, there were more HMGB2- and CMPK1-postive cells in KS lesions than in normal skin tissues (S3 Fig and Fig 3E).

**Table 1:** The partial cellular proteins upregulated >1.5 folds in HUVECs expressing vIRF1.

Protein name	Fold	Protein name	Fold
NAP1L1	3.44 ± 0.05	HMGB2	1.76 ± 0.01
HMGB1	2.45 ± 0.13	PPA1	1.76 ± 0.12
CAST	2.35 ± 0.09	TOP1	1.71 ± 0.09
PTMS	2.21 ± 0.03	CMPK1	1.70 ± 0.09
CROCC	1.94 ± 0.35	DR1	1.68 ± 0.09
PGAM1	1.85 ± 0.01	FASN	1.63 ± 0.06
H1F0	1.81 ± 0.13	DNMT1	1.62 ± 0.22
ST13	1.78 ± 0.01	TXNL1	1.58 ± 0.08

**Fig 4:**
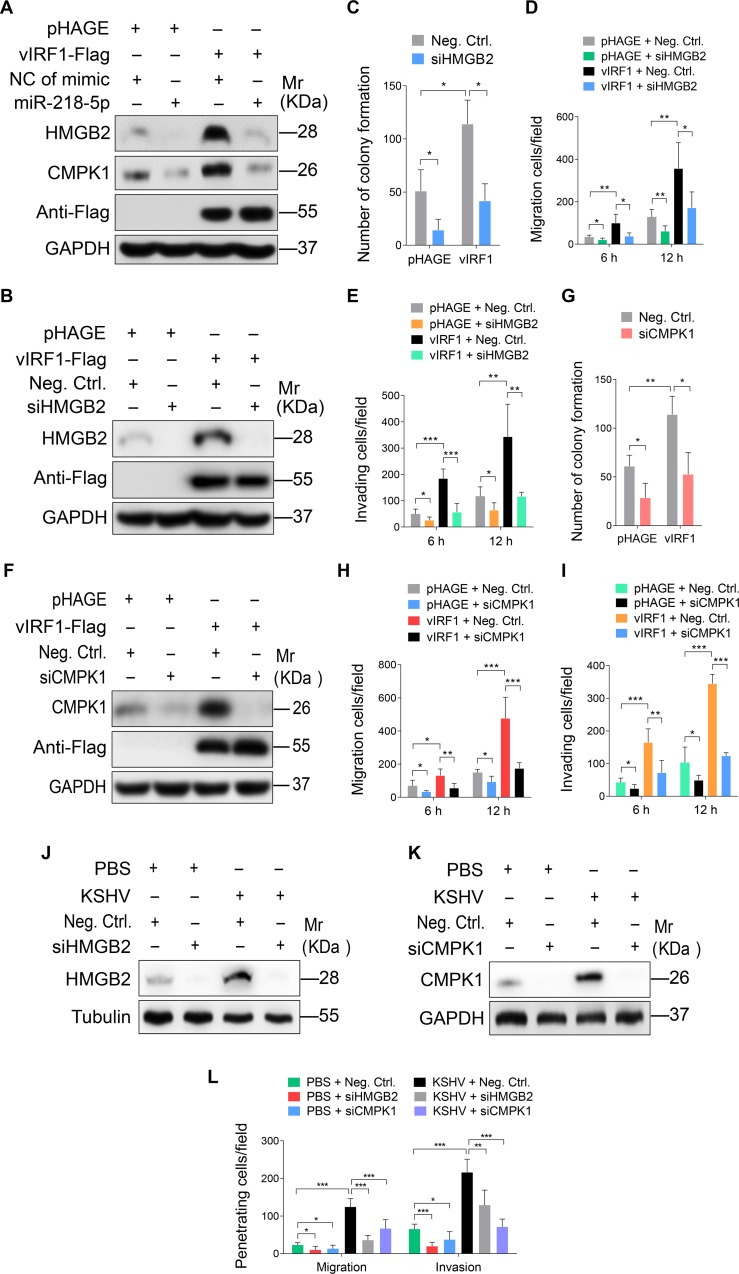
miR-218-5p directly targets HMGB2 and CMPK1 to mediate vIRF1-induced cell motility, invasion and proliferation. **(A).** Western-blotting of HMGB2 and CMPK1 expression in vIRF1-infected HUVECs transfected with a negative control mimic (**NC of mimic**; 20 nM), miR-218-5p mimic (**miR-218-5p**; 20 nM). **(B).** Western-blotting of HMGB2 expression in vIRF1-expressing HUVECs transfected with a mixture of siRNAs targeting HMGB2 (**siHMGB2**). **(C).** Plate colony formation assay of HUVECs treated as in (**B**). **(D).** Migration analysis of HUVECs treated as in (**B**). **(E).** Invasion analysis of HUVECs treated as in (**B**). **(F).** Western-blotting of CMPK1 expression in vIRF1-expressing HUVECs transfected with a mixture of siRNAs targeting CMPK1 (**siCMPK1**). **(G).** Plate colony formation assay of HUVECs treated as in (**F**). **(H).** Migration analysis of HUVECs treated as in (**F**)**. (I).** Invasion analysis of HUVECs treated as in (**F**)**. (J).** Western-blotting of HMGB2 expression in KSHV-infected HUVECs transfected with a mixture of siRNAs targeting HMGB2 (**siHMGB2**). **(K).** Western-blotting of CMPK1 expression in KSHV-infected HUVECs transfected with a mixture of siRNAs targeting CMPK1 (**siCMPK1**). **(L).** Migration and invasion analyses of HUVECs treated as in (**J**) and (**K**) at 6 h. The quantified results represent mean ± SD. Results are from three independent experiments, each with cubic (colony formation) or quadruple (migration and invasion) technical replicates, were performed. * *P* < 0.05, ** *P* < 0.01, and *** *P* < 0.001, Student's t-test.

Previous studies have shown that HMGB2 and CMPK1 are abundantly up-regulated in various malignant tumors, and are closely associated with tumor development and poor prognosis [37–46]. To determine if upregulation of HMGB2 and CMPK1 was necessary for vIRF1-induced cell motility, and proliferation, we silenced HMGB2 and CMPK1 expression in vIRF1-transduced HUVECs with a mixture of siRNAs, respectively (S4 Fig), and observed diminished vIRF1-induced cell migration, invasion and proliferation (Fig 4A–4I). Moreover, knock-down of HMGB2 and CMPK1 also inhibited KSHV-induced cell migration and invasion (Fig 4J–4L).

### vIRF1 suppresses miR-218-5p by inhibiting p53 to increase DNMT1 expression and hypermethylation of the pre-miR-218-1 promoter

**Fig 5:**
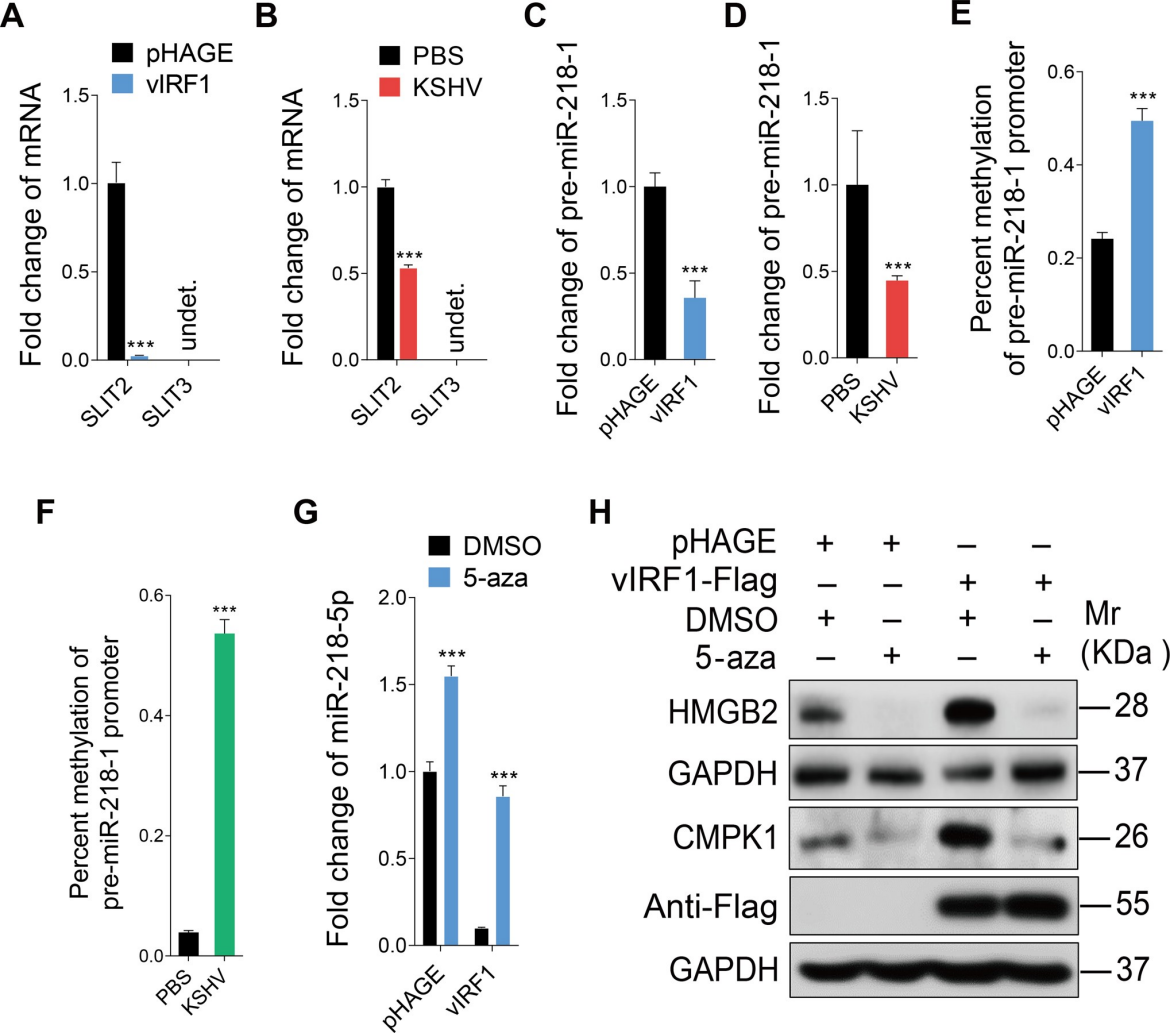
vIRF1 suppresses miR-218-5p via hypermethylation of the pre-miR-218-1 promoter. **(A).** qPCR showing SLIT2 and SLIT3 expression in vIRF1-transduced HUVECs. **(B).** qPCR showing SLIT2 and SLIT3 expression in KSHV-infected HUVECs. **(C).** qPCR showing pre-miR-218-1 expression in vIRF1-transduced HUVECs. **(D).** qPCR showing pre-miR-218-1 expression in KSHV-infected HUVECs **(E).** Methylation-specific PCR showing DNA methylation of in the pre-miR-218-1 promoter in vIRF1-transduced HUVECs. **(F).** Methylation-specific PCR showing DNA methylation of the pre-miR-218-1 promoter in KSHV-infected cells. **(G).** qPCR showing miR-218-5p expression in vIRF1-transduced HUVECs treated with 5-aza. **(H).** Western-blotting of HMGB2 and CMPK1 expression in vIRF1-transduced HUVECs treated with 5-aza. The Western blots were ran with the same samples but in two different gels, with each of them calibrated by independent GAPDH panels. The quantified results represent mean ± SD. *** *P* < 0.001, Student's t-test. undet., undetermined.

MiR-218-5p is expressed from two separate loci, pre-miR-218-1 and pre-miR-218-2, which are co-expressed with their host genes SLIT2 and SLIT3, respectively [47]. The expression of miR-218-5p depends on the promoter activity of its host genes, and hypermethylation of the promoter inhibits miR-218-5p expression [48]. Therefore, we examined the expression of SLIT2 and SLIT3. The expression of SLIT2 mRNA was low in both vIRF1-transduced and KSHV-infected cells while no expression of SLIT3 was detected in HUVECs (Fig 5A and 5B). These results indicated that vIRF1 might suppress miR-218-5p by reducing the expression of primary form pre-miR-218-1 and its host gene SLIT2 by epigenetically silencing their promoter. Indeed, the expression of pre-miR-218-1 was significantly suppressed by both vIRF1 and KSHV infection (Fig 5C and 5D). Consistent with these results, methylation-specific PCR showed that the promoter of pre-miR-218-1 was more hypermethylated in vIRF1-expressing cells and KSHV-infected cells than normal cells (Fig 5E and 5F). Moreover, treatment with a potent inhibitor of DNA methylation, 5-aza, not only blocked vIRF1 suppression of miR-218-5p (Fig 5G), but also decreased the expression of its targets HMGB2 and CMPK1 (Fig 5H). These results revealed that vIRF1 silencing of miR-218-5p expression was due to DNA methylation on its promoter.

**Fig 6:**
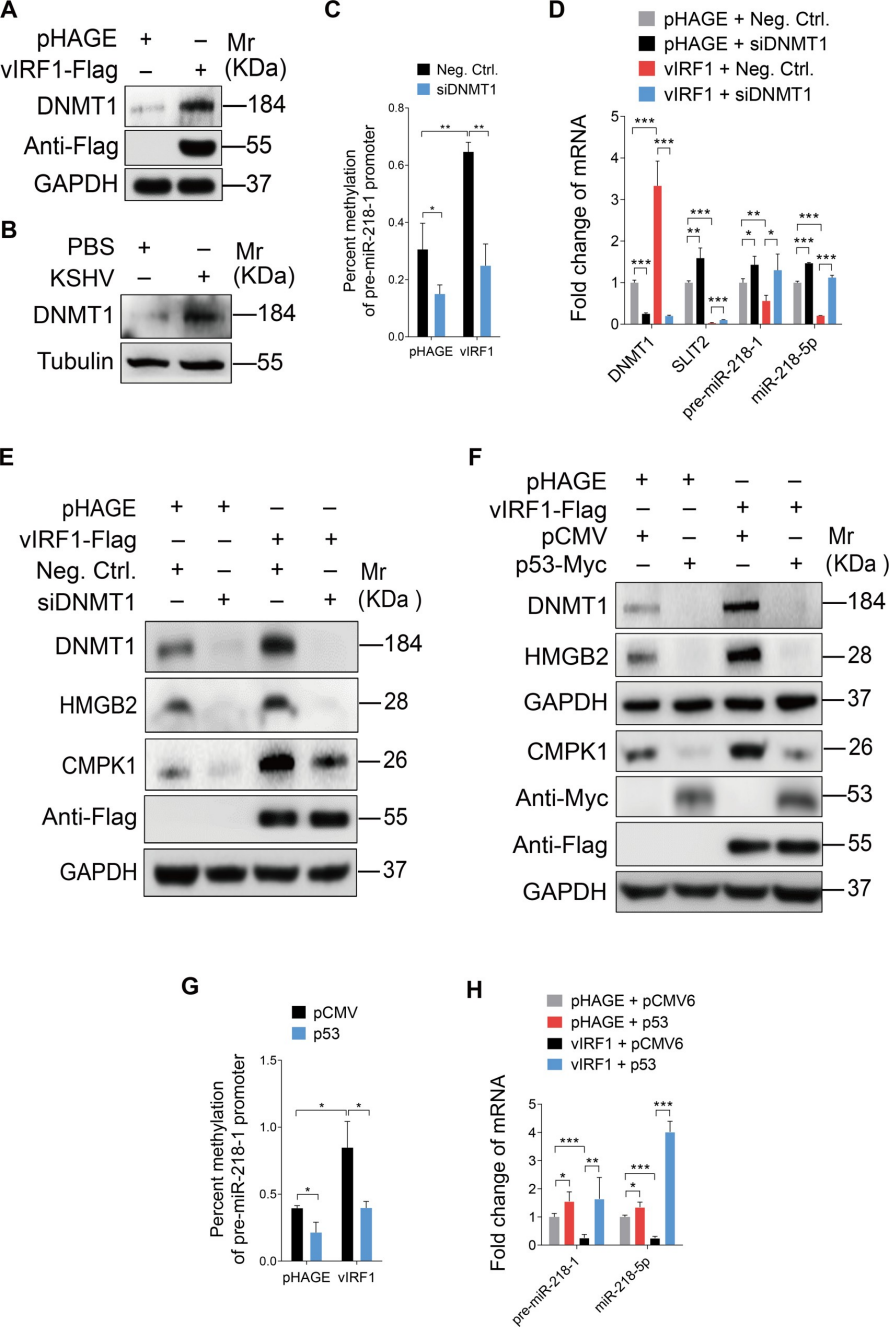
vIRF1 suppresses miR-218-5p via hypermethylation of the pre-miR-218-1 promoter by inhibiting p53 to increase DNMT1 expression. **(A).** Western-blotting of DNMT1 expression in vIRF1-transduced HUVECs. **(B).** Western-blotting of DNMT1 in KSHV-infected HUVECs. **(C).** Methylation-specific PCR showing DNA methylation of the pre-miR-218-1 promoter in vIRF1-expressing HUVECs transfected with a mixture of siRNAs targeting DNMT1 (**siDNMT1**). **(D).** qPCR showing DNMT1, SLIT2, pre-miR-218-1 and miR-218-5p expression in vIRF1-expressing HUVECs transfected with a mixture of siRNAs targeting DNMT1 (**siDNMT1**). **(E).** Western-blotting of DNMT1, HMGB2 and CMPK1 expression in vIRF1-expressing HUVECs transfected with a mixture of siRNAs targeting DNMT1 (**siDNMT1**). **(F).** Western-blotting of DNMT1, HMGB2 and CMPK1 expression in vIRF1-expressing HUVECs transfected with pCMV6-Entry-C-Myc-p53 construct. The Western blots were ran with the same samples but in two different gels, with each of them calibrated by independent GAPDH panels. **(G).** Methylation-specific PCR showing DNA methylation of the pre-miR-218-1 promoter in vIRF1-expressing HUVECs transfected with pCMV6-Entry-C-Myc-p53 construct. **(H).** qPCR showing pre-miR-218-1 and miR-218-5p expression in vIRF1-expressing HUVECs transfected with pCMV6-Entry-C-Myc-p53 construct. The quantified results represent mean ± SD. * *P* < 0.05, ** *P* < 0.01, and *** *P* < 0.001, Student's t-test.

DNA methyltransferase 1 (DNMT1) mediates DNA methylation and has been reported to cause miR-218-5p silencing [49]. We found that DNMT1 was up-regulated by 1.62-fold in vIRF1-transduced HUVECs (Table 1). Moreover, Western-blotting confirmed that DNMT1 protein was up-regulated in vIRF1-expressing cells and KSHV-infected cells (Fig 6A and 6B). Knock-down of DNMT1 expression with specific siRNAs (siDNMT1) reduced the hypermethylation of the promoter of pre-miR-218-1 in vIRF1 expressing cells (Fig 6C), and enhanced the expression of SLIT2, pre-miR-218-1 and miR-218-5p (Fig 6D). Meanwhile, vIRF1 induced expression of HMGB2 and CMPK1 was also abolished following DNMT1 inhibition (Fig 6E).

vIRF1 binds to p53 and represses p53-dependent transcription and apoptosis [16]. p53 transcriptionally suppresses the DNMT1 promoter by interacting with specificity protein 1 (Sp1) and forma complex [50]. Based on these studies, we sought to elucidate whether vIRF1 inhibition of p53-dependent transcription was responsible for vIRF1-induced DNMT1 up-regulation and therefore was involved in the inhibition of miR-218-5p. Indeed, overexpression of p53 in vIRF1-transduced HUVECs reduced the expression levels of DNMT1, HMGB2 and CMPK1 (Fig 6F), as well as hypermethylation of the promoter of pre-miR-218-1 in vIRF1-expressing cells (Fig 6G), hence causing an increase of both pre-miR-218-1 and miR-218-5p expression in vIRF1-infected HUVECs (Fig 6H).

These results indicated that vIRF1-induced miR-218-5p inhibition via aberrant DNA methylation at the pre-miR-218-1 promoter by inhibiting p53 to cause DNMT1 upregulation.

### The crosstalk between miR-218-5p and lncRNA-OIP5-AS1 contributes to vIRF1-induced cell motility, and proliferation

**Fig 7:**
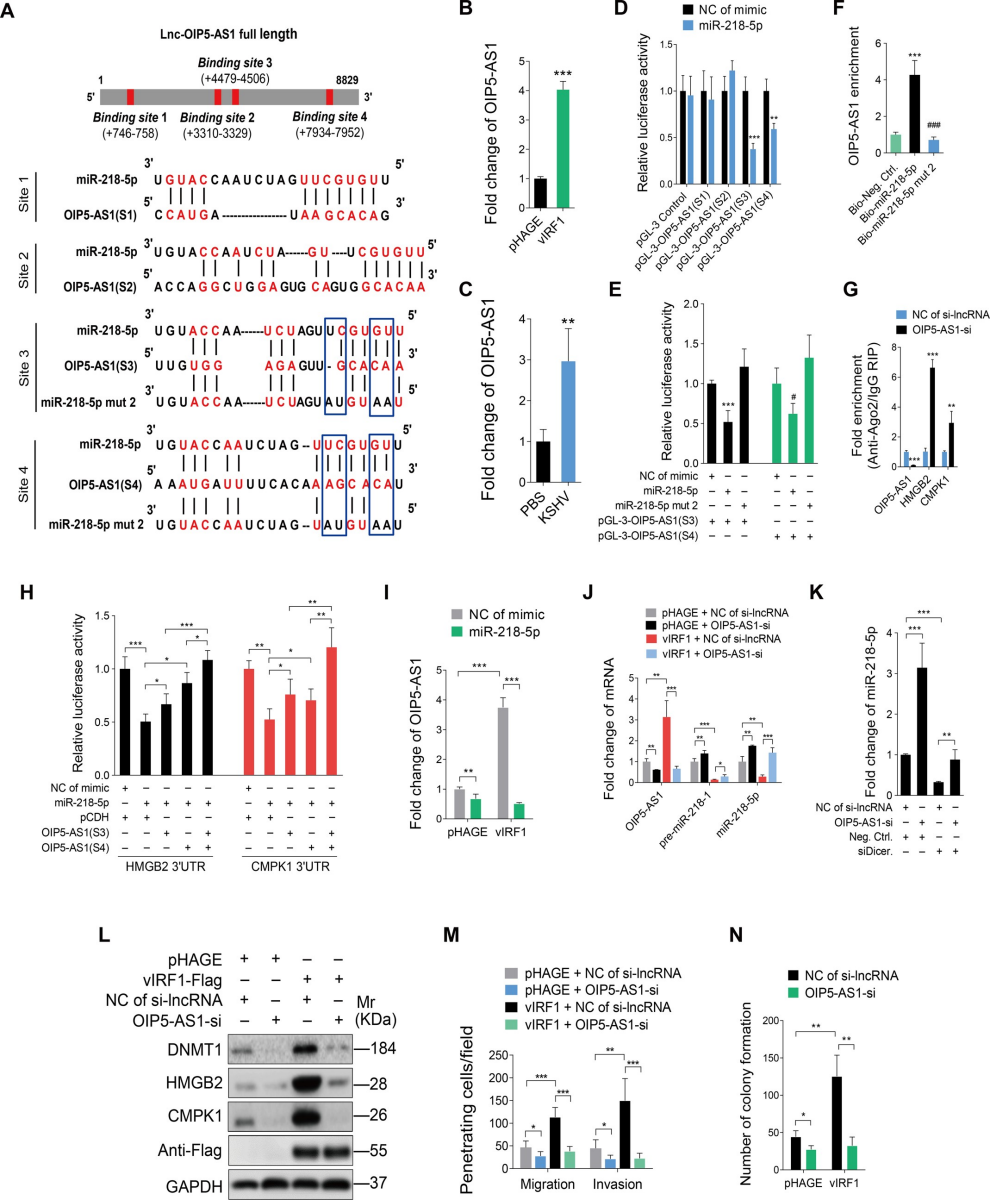
The crosstalk between miR-218-5p and lncRNA-OIP5-AS1 contributes to vIRF1-induced cell motility, invasion and proliferation. **(A).** Putative binding site of miR-218-5p in lnc-OIP5-AS1 and mutagenesis of target sites in miR-218-5p. **(B).** qPCR showing lncRNA-OIP5-AS1 expression in vIRF1-transduced HUVECs. **(C).** qPCR showing lnc-OIP5-AS1 expression in KSHV-infected HUVECs. **(D).** Luciferase activity in HEK293T cells cotransfected with miR-218-5p mimic (**miR-218-5p**; 20 nM) or a negative control (**NC of mimic**; 20 nM), together with the pGL-3-OIP5-AS1(S1), pGL-3-OIP5-AS1(S2), pGL-3-OIP5-AS1(S3) or pGL-3-OIP5-AS1(S4) reporter construct for 48 h. **(E).** Luciferase activity in HEK293T cells cotransfected with the miR-218-5p mimic (**miR-218-5p**; 20 nM), miR-218-5p mutant mimic (**miR-218-5p mut 2**; 20 nM) or a negative control (**NC of mimic**; 20 nM) together with the pGL-3-OIP5-AS1(S3), or pGL-3-OIP5-AS1(S4) reporter construct for 48 h. **(F).** RNA pull-down analysis was performed with biotin-labeled miRNA and its control including bio-Neg. Ctrl., bio-miR-218-5p and bio-miR-218-5p mut 2. Specific primers were used to detect the enrichment of lnc-OIP5-AS1. **(G).** RIP results showing the enrichment of binding of lnc-OIP5-AS1, HMGB2 and CMPK1 with miR-218-5p based on Ago2 pull down assay. **(H).** Luciferase activity in HEK293T cells cotransfected with miR-218-5p mimic (**miR-218-5p**; 20 nM) or a negative control (**NC of mimic**; 20 nM) together with pGL3-HMGB2 3'UTR reporter or pGL3-CMPK1 3'UTR reporter with and without lnc-OIP5-AS1(S3) or lnc-OIP5-AS1(S4) fragment. **(I).** qPCR showing lnc-OIP5-AS1 expression in vIRF1-expressing HUVECs transfected with miR-218-5p mimic. **(J).** qPCR showing lnc-OIP5-AS1, pre-miR-218-1 and miR-218-5p expression in vIRF1-expressing HUVECs with lnc-OIP5-AS1 silencing. **(K).** qPCR showing miR-218-5p expression in lnc-OIP5-AS1 silenced HUVECs with Dicer knockdown. **(L).** Western-blotting of DNMT1, HMGB2 and CMPK1 expression in vIRF1 cells with lnc-OIP5-AS1 silencing. **(M).** Migration and invasion analyses of vIRF1 cells with lnc-OIP5-AS1 silencing at 6 h. **(N).** Plate colony formation assay of vIRF1-transduced HUVECs with lnc-OIP5-AS1 silencing. The quantified results represent mean ± SD. * *P* < 0.05, ** *P* < 0.01, *** *P* < 0.001, and ^###^ *P* < 0.001, Student's t-test.

Numerous studies have shown that lncRNAs can act as ceRNAs to regulate the functions of miRNAs. To identify lncRNAs which may serve as ceRNAs and interact with miR-218-5p, we utilized online software programs starbase v2.0 (http://starbase.sysu.edu.cn/) and LncBase Predicted v.2 (http://carolina.imis.athena-innovation.gr/diana_tools/web/index.php?r=lncbasev2%2Findex-predicted) to search for lncRNAs that have complementary base pairing with miR-218-5p. Considering the abundance in the cytoplasm, and high score of predicted binding sites, we identified lncRNA OIP5 antisense RNA 1 (lncRNA-OIP5-AS1) as a potential candidate. We found that there were four putative miR-218-5p-binding sites in lnc-OIP5-AS1 (Fig 7A) and that lnc-OIP5-AS1 was indeed up-regulated in both vIRF1-transduced and KSHV-infected HUVECs (Fig 7B and 7C). We also found that vIRF1 was capable of activating the luciferase activity of lnc-OIP5-AS1 promoter (S5 Fig). We generated four luciferase reporter constructs, each of which contains only one putative miR-218-5p-binding site. Of these, miRNA-218-5p mimics reduced the luciferase activities of lnc-OIP5-AS1 (S3) and lnc-OIP5-AS1 (S4) reporters (Fig 7D) in a dose-dependent fashion (S6 Fig). In contrast, the miR-218-5p mutant mimic lacking the seed sequence did not reduce the luciferase activities of both lnc-OIP5-AS1 (S3) and lnc-OIP5-AS1 (S4) reporters (Fig 7E). To confirm the physical interaction between miR-218-5p and lnc-OIP5-AS1, we performed RNA pull-down experiments. Biotin-labeled mimics were incubated with HUVECs lysates, isolated with streptavidin agarose beads and then analyzed by RT-qPCR. We observed that lnc-OIP5-AS1 enriched miR-218-5p but not the miR-218 mutant (Fig 7F). These results indicated that lnc-OIP5-AS1 could directly bind miR-218-5p.

To determine whether lnc-OIP5-AS1 could act as a ceRNA to abrogate the function of miR-218-5p by releasing its binding to the targeted transcripts, we knocked down lnc-OIP5-AS1 with specific Smart Silencer in HUVECs and performed an RNA immunoprecipitation (RIP) assay on Ago2 (S7 Fig). We found that silencing of lnc-OIP5-AS1 in HUVECs increased the HMGB2 and CMPK1 transcripts in the Ago2 complex (Fig 7G). Furthermore, overexpression of lnc-OIP5-AS1 (S3) and lnc-OIP5-AS1 (S4) fragments abolished the inhibition of HMGB2 and CMPK1 3’UTR reporter activities by miR-218-5p (Fig 7H). These results demonstrated the sequestration of miR-218-5p by lnc-OIP5-AS1, which relieved the inhibition of the HMGB2/CMPK1 transcripts by miR-218-5p.

Intriguingly, overexpression of miR-218-5p significantly reduced the level of vIRF1-induced lnc-OIP5-AS1 (Fig 7I). Conversely, inhibition of lnc-OIP5-AS1 reversed vIRF1 inhibition of the expression of both pre-miR-218-1 and miR-218-5p (Fig 7J). We further performed knock down of Dicer to prevent maturation of miR-218-5p from pre-miR-218-1 and then examined the effect of silencing lnc-OIP5-AS1 on miR-218-5p stability (S8 Fig). We found that suppression of lnc-OIP5-AS1 reduced the degradation of miR-218-5p (Fig 7K). As a result, silencing of lnc-OIP5-AS1 attenuated vIRF1-induced DNMT1, HMGB2 and CMPK1 expression (Fig 7L). These results indicated that lnc-OIP5-AS1 could not only inhibit the function of miR-218-5p by acting as a ceRNA but also reduce the level of miR-218-5p by direct binding to induce miR-218-5p degradation. Consistent with these results, silencing of lnc-OIP5-AS1 inhibited vIRF1-induced cell migration, invasion and proliferation (Fig 7M and 7N).

### Loss of vIRF1 reduces KSHV-induced cell motility

**Fig 8:**
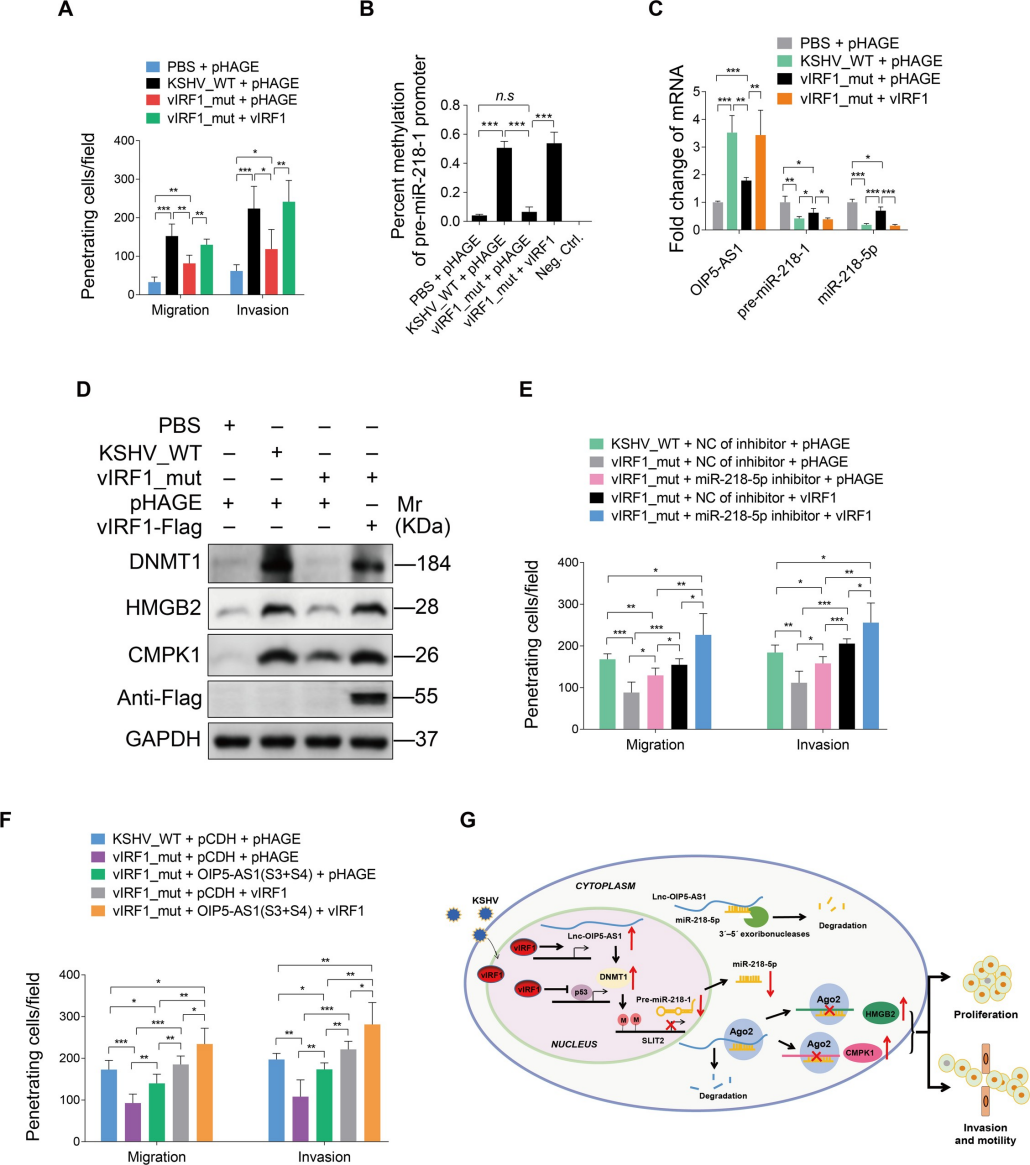
Loss of vIRF1 reduces cell motility, invasion and proliferation induced by KSHV. **(A).** Migration and invasion analyses of HUVECs treated with PBS (**PBS**) or infected with wild-type KSHV (**KSHV_WT**) or vIRF1 mutant virus (**vIRF1_mut**) followed by transduction with lentiviral vIRF1 at MOI 2 at 6 hpi. **(B).** Methylation-specific PCR showing DNA methylation of HUVECs treated as in (**A**). **(C).** Expression of lnc-OIP5-AS1, pre-miR-218-1 and miR-218-5p expression detected by qPCR in HUVECs treated as in (**A**). **(D).** Western-blotting of DNMT1, HMGB2 and CMPK1 expression in HUVECs treated as in (**A**). **(E).** Migration and invasion analyses of wild-type KSHV (**KSHV_WT**) cells, vIRF1 mutant cells (**vIRF1_mut**) or vIRF1-transduced mutant cells followed by transduction with a specific miR-218-5p inhibitor at 6 hpi. **(F).** Migration and invasion analyses of wild-type KSHV (**KSHV_WT**) cells, vIRF1 mutant cells (**vIRF1_mut**) or vIRF1-transduced mutant cells followed by transduction with lentiviral lnc-OIP5-AS1 (S3) construct and lentiviral lnc-OIP5-AS1 (S4) construct at 6 h. **(G).** A hypothetical model of the mechanism of how vIRF1 facilitates endothelial cell motility, invasion and proliferation. The quantified results represent mean ± SD. * *P* < 0.05, ** *P* < 0.01, and *** *P* < 0.001, Student's t-test. *n*.*s*, not significant.

To further dissect the functions of vIRF1 in the context of KSHV genome, we constructed a KSHV mutant with ORF-K9 deleted using a two-step red recombination system as previously described [51–53]. Positive colonies were screened and verified by PCR (S9A and S9B Fig). Restriction analysis showed that the RGB-K9-mutant had a band shift of about 1.3 kb compared to the wild-type RGB-BAC16, indicating that the K9 mutant bacmid was successfully generated (S9C Fig). The RGB-K9-mutant was transfected into iSLK cells and selected to obtain stable producer iSLK cells. As expected, we did not detect the expression of vIRF1 in iSLK-RGB-K9 mutant cells, while the levels of vIRF4 and ORF57 had minimal changes (S9D Fig). Similarly, HUVECs infected by the mutant virus had no vIRF1 expression (S9E Fig) but had minimal changes in the levels of vIRF4 and ORF57 (S9F Fig). As expected, expression levels of phosphorylated p53, acetylated p53, and p21 were increased in vIRF1_mutant cells compared to those of KSHV_WT virus-infected HUVECs (S9G Fig). Because HUVECs transduced with lentiviral vIRF1 at a MOI of 2 showed a vIRF1 mRNA expression level similar to that of wild type KSHV-infected HUVECs (S1 Fig), we transduced vIRF1_mutant cells with 2 MOI of lentiviral vIRF1. We found that loss of vIRF1 not only reduced cell migration and invasion (Fig 8A) but also decreased the level of hypermethylation in the pre-miR-218-1 promoter (Fig 8B). Importantly, complementation with vIRF1 in vIRF1_mut-infected HUVECs was sufficient to rescue cell migration and invasion induced by KSHV (Fig 8A), and reverse the level of hypermethylation in the pre-miR-218-1 promoter induced by KSHV (Fig 8B). We also observed a significant decrease of lnc-OIP5-AS1 expression, and an increase of miR-218-5p and pre-miR-218-1 expression in the mutant cells compared to both KSHV_wild-type infected cells and vIRF1-transduced mutant cells (Fig 8C). Furthermore, deletion of vIRF1 reduced the expression of DNMT1, HMGB2 and CMPK1 while complementation with vIRF1 was sufficient to rescue the expression levels of these proteins (Fig 8D). Meanwhile, inhibition of miR-218-5p with a specific inhibitor in both mutant cells and vIRF1-transduced mutant cells increased cell migration and invasion (Fig 8E). Similar increase in cell migration and invasion was also observed in the mutant and vIRF1-transduced mutant cells following overexpression of lnc-OIP5-AS1 (S3) and lnc-OIP5-AS1 (S4) fragments (Fig 8F). Taken together, these results demonstrated that vIRF1 mediated KSHV-induced cell migration, and invasion by down-regulating miR-218-5p and up-regulating lnc-OIP5-AS1.

## Discussion

KSHV K9/vIRF1 was initially characterized as an early lytic gene. However, subsequent studies have shown that it is also expressed during viral latency. vIRF1 has two transcription start sites, one is distal to the AUG, which is active during latency in PEL, and another is a more proximal site, which is induced upon lytic reactivation [54]. Hence, vIRF1 might have a dual modes of expression during latent and lytic replication [55–57]. Furthermore, K9/vIRF1 mRNA is expressed in all KS tumors (total 21 KS clinical biopsies) and preferentially transcribed during latent infection of either endothelial/mesenchymal lineage cells, which strengthens the role of K9/vIRF1 in KS tumorigenesis [58]. In the current study, we found that vIRF1 promoted endothelial cell migration and invasion, as well as proliferation. Further, deletion of vIRF1 from the KSHV genome reduced KSHV-induced cell migration, invasion and proliferation. However, we could not assess the expression level of the endogenous vIRF1 protein because there is currently no vIRF1 antibody available. Despite the limitation, this work still revealed a novel role of vIRF1 in cell migration, invasion and proliferation, which is an important part of KS pathogenesis, particularly in the invasiveness and dissemination of KS tumors.

MiR-218-5p, a vertebrate-specific intronic miRNA co-regulated with its host genes SLIT2/SLIT3, functions as a tumor suppressor by modulating multiple pathways [36]. It is downregulated in numerous human cancers, such as colorectal, prostate, pancreatic, gastric, and thyroid cancers [47, 49, 59–63]. The mechanism of silencing miR-218-5p and its host genes, SLIT2/SLIT3 is through promoter hypermethylation. For instance, human papillomavirus type 16 oncogene E6 reduces the level of miR-218-5p and SLIT2 through promoter hypermethylation [64]. However, significance of miR-218-5p in the development of KS remains undefined. In this study, we revealed that both KSHV infection and vIRF1 expression reduced the level of miR-218-5p at least in part by silencing of pre-miR-218-1/SLIT2 via promoter hypermethylation. Further, we demonstrated that vIRF1 increased DNMT1 expression by inhibiting p53 transcriptional activity, leading to a higher level of DNA methylation of the pre-miR-218-1 promoter.

Lnc-OIP5-AS1 located at chromosome 15q15.1, known as cyrano, is ~8,000 nucleotides in length and abundant in the cytoplasm. It was originally characterized in zebrafish and displayed crucial effects in embryonic nervous system development [65]. It was also reported to play a vital role in embryonic stem cells (ESCs) self-renewal maintenance [66]. With regard to its role in cancer, lnc-OIP5-AS1 exhibits multifaceted and complex features. For example, lnc-OIP5-AS1 is shown to be a tumor suppressor and inhibit HeLa cells proliferation by interacting with the RBP HuR to reduce HuR’s availability for binding target mRNAs, or associating with GAK mRNA to impair GAK mRNA stability [67, 68]. On the contrary, lnc-OIP5-AS1 can exert oncogenic functions in several other cancers. It was consistently up-regulated in renal cell carcinoma, glioblastoma, and gastric cancer [69–71]. Silencing of lnc-OIP5-AS1 repressed YAP-Notch signaling pathway activity leading to decrease of glioma cells’ proliferation, migration *in vitro* and tumor formation *in vivo* [72]. Moreover, lnc-OIP5-AS1 was highly expressed in lung adenocarcinoma tissues and cells, and the loss of lnc-OIP5-AS1 inhibited lung adenocarcinoma cell proliferation, migration and invasion [73]. In our report, we found that both KSHV infection and vIRF1 expression increased the expression of lnc-OIP5-AS1 in endothelial cells. Silencing of lnc-OIP5-AS1 suppressed cell migration, invasion and proliferation. Intriguingly, we found that vIRF1 activated the transcription of lnc-OIP5-AS1, however, the precise mechanism remains unknown.

The cross-regulatory interactions between lncRNAs and miRNAs have been recognized to regulate their downstream targets of either lncRNAs or miRNAs [74, 75]. Several miRNAs including miR-7 [66], miR-410 [76], miR-424 [67], and miR-448 [73] have been identified to interact with lnc-OIP5-AS1. On the other hand, miR-218-5p has been reported to interact with lnc-MALAT1, participating in choriocarcinoma growth [77]. In the current study, we revealed the crosstalk between miR-218-5p and lnc-OIP5-AS1, confirmed a direct interaction between miR-218-5p and lnc-OIP5-AS1, and unearthed the fateful consequences of this interaction. We showed that lnc-OIP5-AS1 functioned as a ceRNA and sequestered miR-218-5p to relieve its binding and targeting of HMGB2 and CMPK1 transcripts. Further, lnc-OIP5-AS1 could inhibit miR-218-5p expression through regulating miR-218-5p stability. Once entering the RNA-induced silencing complex (RISC), miRNAs become extremely stable due to the protection of both ends of miRNAs by AGO proteins from 3ʹ–5ʹ exoribonucleases-mediated degradation. Therefore, we speculated that lnc-OIP5-AS1 might block miR-218-5p from loading onto AGO proteins, and hence accelerate its degradation. Interestingly, by an unclear mechanism, lnc-OIP5-AS1 also increased DNMT1 expression to promote DNA methylation of the pre-miR-218-1 promoter, leading to decreased level of miR-218-1. On other hand, the lnc-OIP5-AS1/miR-218-5p interaction also resulted in miR-218-5p suppression of lnc-OIP5-AS1 expression albeit the precise mechanism is unknown.

In conclusion, our study revealed that vIRF1 promoted cell migration, invasion and proliferation by a p53- and lnc-OIP5-AS1-mediated down-regulation of miR-218-5p, leading to increased expression levels of its target genes HMGB2 and CMPK1 (Fig 8G). This process was mediated by the complex crosstalk between miR-218-5p and lnc-OIP5-AS1. These novel findings extend the cross-regulatory network of cellular lncRNAs and miRNAs involved in the pathogenesis of KS.

## Materials and methods

### Ethics statement

The clinical section of the research was reviewed and ethically approved by the Institutional Ethics Committee of the First Affiliated Hospital of Nanjing Medical University (Nanjing, China; Study protocol # 2015-SR-116). Written informed consent was obtained from all participants, and all samples were anonymized. All participants were adults.

### Cell culture and plasmids

The iSLK cells were cultured in Dulbecco's modified Eagle's medium (DMEM) supplemented with 1% penicillin-streptomycin, 1 μg/ml puromycin and 250 μg/ml G418. The established iSLK-RGB-BAC16 and iSLK-RGB-K9 mutant cells were cultivated in DMEM supplemented with 10% fetal bovine serum (FBS), 1 μg/ml puromycin, 250 μg/ml G418, and 1.2 mg/ml hygromycin B [53]. HEK293T and continuous cell lines human umbilical vein endothelial cells (catalog #CRL-1730, ATCC, Manassas, VA, USA) were maintained as previously described [78]. The latter were only used for plate colony formation assay to evaluate the ability of cell proliferation. Primary human umbilical vein endothelial cells (HUVECs), which were used for all assays except for luciferase and plate colony formation assays, were isolated and cultured as previously delineated [79].

Flag-vIRF1 was cloned by inserting the coding sequences into plasmid pHAGE-CMV-MCSIzsGreen as previously described [78, 80]. The respective sequences of HMGB2 3’UTR, CMPK1 3’UTR and lncRNA-OIP5-AS1 fragments containing putative miR-218-5p binding sites (S1: 365–1167; S2: 3078–3539; S3: 4293–4701 and S4: 7775–8169) were amplified by PCR and inserted into the pGL3-Control plasmid (Promega, Madison, WI, USA), respectively. The pCMV6-Entry-C-Myc-p53 construct and pCMV6-Entry-C-Myc construct were provided by ORIGENE (Beijing, China). The DNA fragment of lnc-OIP5-AS1 covering –1500 bp to 0 bp of the transcription start site was amplified and subcloned into pGL3-Basic plasmid (Promega, Madison, WI, USA).

### Transfection, Reagents, Antibodies, and Western-Blotting

HUVECs were transfected using the Effectence transfection reagent (Qiagen, Suzhou, Jiangsu, China), while HEK293T cells were transfected using the Lipofectamine 2000 Reagent (Invitrogen, Carlsbad, CA, USA). 5-aza (Decitabine), a potent inhibitor of DNA methylation, was from Selleck Chemicals (Shanghai, China). siRNAs were synthesized from Genepharma (Suzhou, China), the sequences of siRNAs are listed in S1 Table. LncRNA Smart Silencer was obtained from RiboBio (Guangzhou, China). Antibodies against KSHV LANA, HMGB2, CMPK1, DNMT1 and Dicer were from Abcam (Cambridge, MA, USA). Anti-Flag was obtained from Cell Signaling Technologies (Beijing, China). Anti-Myc, anti-α-Tubulin, and anti-GAPDH were from Santa Cruz Biotechnology (Dallas, TX, USA). Anti-rabbit immunoglobulin G (IgG), anti-mouse IgG, anti-phosphorylated p53, anti-acetylated p53, anti-p53, and anti-p21 were purchased from Beyotime Institute of Biotechnology (Nantong, Jiangsu, China). Western-blotting analysis was conducted as previously described [81, 82]. In this study, all Western blotting results were independently repeated at least three times unless otherwise stated.

### Cell migration, invasion and plate colony formation assays

Cell migration, invasion and colony formation assays were executed as previously described [83–85].

### Luciferase reporter assay

Luciferase reporter assay was conducted using the Promega dual-luciferase reporter assay system according to the previous study [86].

### Methylation-Specific PCR (MS-PCR)

Methylation-specific PCR (MS-PCR) was adopted using DNA Bisulfite conversion kit (TIANGEN BIOTECH, Beijing, China) and Methylation-specific PCR kit (TIANGEN BIOTECH, Beijing, China) according to the manufacturer’s instructions. MS-PCR primers were designed as previously described [87].

### RNA pull-down assay

HUVECs were collected, washed, and re-suspended with lysis buffer (Thermo Fisher Scientific, Waltham, America). After incubating for 5 min, the lysates were precleared by centrifugation at 14,000 rpm for 10 minutes, and then were added to streptaviden magnetic beads (Thermo Fisher Scientific, Waltham, America), which were incubated with Biotin-labeled miR-218-5p, miR-218-5p mut 2, or Neg. Ctrl (Genepharma, Suzhou, China) for 4 hours. The bound RNAs in the pull-down material were quantified by qRT-PCR.

### RNA Immunoprecipitation (RIP) assay

HUVECs were transfected with lnc-OIP5-AS1 Smart Silencer or its Neg. Ctrl for 48 h, and used for RIP experiments with an anti-Ago2 antibody (MERCK, Darmstadt, Germany) and the Magna RIPTM RNA-Binding Protein Immunoprecipitation Kit (MERCK, Darmstadt, Germany), according to the manufacturer’s instructions. The levels of lnc-OIP5 AS1, HMGB2 or CMPK1 were examined by qRT-PCR.

### Construction and identification of KSHV ORF K9 Mutant

A KSHV mutant with ORF K9 deleted was constructed as described in previous studies [52, 88]. In brief, using the bacterial artificial chromosome (BAC) technology and the *Escherichia coli* Red recombination system, together with PCR, restriction digestion, and sequencing for strict quality control, a KSHV ORF K9 mutant (called RGB-K9-mutant) was constructed by removing K9 coding sequence (CDS) from the wild-type recombinant KSHV RGB-BAC16 [53]. RGB-BAC16 and RGB-K9 mutant DNA were transfected into iSLK cells and selected using 1 μg/ml puromycin, 250 μg/ml G418, and 1.2 mg/ml hygromycin B for 3 weeks to establish stable viral producer cell lines, iSLK-RGB-BAC16 and iSLK-RGB-K9 mutant cells. To produce virus stocks for infection, iSLK-RGB-BAC16 and iSLK-RGB-K9 mutant cells were plated at 30 to 40% confluence and induced with both Doxycycline (Dox) (1 μg/ml) and sodium butyrate (NaB) (1 mM). After induction for 4 or 5 d, the supernatant was harvested, centrifuged, filtered, and concentrated by ultracentrifugation (25, 000 g at 4°C for 3 h) using SW32 Ti rotor (Beckman Coulter Inc, USA). The pellet was resuspended, supplemented with 8 μg/mL polybrene and then incubated with 10^5^ HUVECs in a 6-well plate for 4 h. The primers for construction and identification of K9 mutant bacmid were designed as previously described [89] and the sequences of the primers could be found in S2 Table.

### Reverse transcription and real time quantity PCR

RNA was extracted using RNA Isolator Total RNA Extraction Reagent (Vazyme Biotech Co., Ltd, Nanjing, China) from cells. Total RNA was reverse transcription by HiScript Q RT SuperMix (Vazyme Biotech Co., Ltd, Nanjing, China). Real time quantity PCR was performed by AceQ qPCR SYBR Green Master Mix (Vazyme Biotech Co., Ltd, Nanjing, China). The sequences of the primers for PCR could be found in S3 Table.

### The extraction of genome DNA

The extraction of genome DNA was performed by using TIANamp Genomic DNA Kit (TIANGEN BIOTECH, Beijing, China) according to the user’s guide. Briefly, cells were trypsinized, and neutralized by 20% FBS DMEM. The suspension was centrifuged and the supernatant was discarded.

### Mass spectrometry analysis

Mass spectrometry analysis was adopted according to the previous study [86].

### Immunohistochemistry (IHC)

The KS clinical specimens were kindly offered by Jiangsu Province Hospital. All samples were anonymized and all participants are provided with informed consent. IHC was carried out as previously described with specific antibodies [85, 90].

### Statistical analysis

All data are appeared as the means ± SD with at least three replications. Statistical analysis was on account of Student’s *t*-test and the criterion for statistical significance was adopted as *P* values of < 0.05.

### Accession numbers

Microarray data have been submitted and can be accessed by GEO accession number GSE119034.

## Supporting information

S1 Table A list of sequences of the siRNAs mentioned in the text. (DOCX)

S2 Table A list of sequences of primers for deletion and test of ORF-K9 mutagenesis mentioned in the text. (DOCX)

S3 Table A list of sequences of specific primers for qPCR mentioned in the text. (DOCX)

S1 Fig Determination of transduction efficiency of lentivirus-mediated vIRF1 in endothelial cells. qPCR results showing vIRF1 mRNA expression in HUVECs infected with KSHV or transduced with different MOI of lentiviral vIRF1. The level of vIRF1 mRNA in KSHV cells was set as ‘‘1” for comparison. The quantified results represent the mean ± SD.
(TIF)

S2 Fig miR-218-5p overexpression reduces cell migration and invasion. Representative images of migration and invasion analysis of vIRF1-infected HUVECs transfected with mimics of miR-218-5p for 48 h. Original magnification, ×100.
(TIF)

S3 Fig Immunohistochemical staining of KS lesion and normal skin. Immunohistochemical staining of isotype control immunoglobulin G (IgG) for HMGB2 (**Isotype IgG of HMGB2**) and CMPK1 (**Isotype IgG of CMPK1**) in normal skin, and skin KS of patient #2 (**Skin KS2**). Magnification, ×200, ×400.
(TIF)

S4 Fig Knockdown of HMGB2 and CMPK1 with siRNAs. **(A).** Western-blotting of HMGB2 in HUVECs transfected with No.1 (**si1HMGB2**), No. 2 (**si2HMGB2**), No. 3 (**si3HMGB2**), and a mixture of No. 1, 2 and 3 (**siHMGB2 Mix**) siRNAs targeting HMGB2.
**(B).** Western-blotting of CMPK1 in HUVECs transfected with No.1 (**si1CMPK1**), No. 2 (**siCMPK1**), No. 3 (**si3CMPK1**), and a mixture of No. 1, 2 and 3 (**siCMPK1 Mix**) siRNAs targeting CMPK1.
(TIF)

S5 Fig vIRF1 increases the luciferase activity of the lnc-OIP5-AS1 promoter reporter. Luciferase activity in HEK293T cells cotransfected with vIRF1 and the lnc-OIP5-AS1 promoter reporter for 4 h, 6 h, 12 h and 24 h, respectively. The quantified results represent mean ± SD. * *P* < 0.05, *** *P* < 0.001, Student's t-test. *n*.*s*, not significant.
(TIF)

S6 Fig miR-218-5p reduces the luciferase activities of lnc-OIP5-AS1 (S3) and lnc-OIP5-AS1 (S4) reporters in a dose-dependent fashion. Luciferase activity in HEK293T cells cotransfected with an incremental amount of miR-218-5p mimic (**miR-218-5p**) (5, 10, and 15 nM) or its control (**NC of mimic**) together with pGL-3-OIP5-AS1(S3) or pGL-3-OIP5-AS1(S4) reporter for 48 h. The quantified results represent mean ± SD. * *P* < 0.05, ** *P* < 0.01, Student's t-test.
(TIF)

S7 Fig Knockdown of lnc-OIP5-AS1 with specific lncRNA Smart Silencer. qPCR showing lnc-OIP5-AS1 expression in HUVECs transfected with an incremental amount of lncRNA Smart Silencer targeting lnc-OIP5-AS1 (**OIP5-AS1-si**) (50 and 200 nM) for 48 h. Three specific primers of lnc-OIP5-AS1 were used. The quantified results represent mean ± SD. *** *P* < 0.001, Student's t-test.
(TIF)

S8 Fig Knockdown of Dicer with siRNAs. Western-blotting of Dicer in HUVECs transfected with No.1 (**si1Dicer**), No. 2 (**si2Dicer**), No. 3 (**si3Dicer**), and a mixture of No. 1, 2 and 3 (**siDicer Mix**) siRNAs targeting Dicer.
(TIF)

S9 Fig Construction and identification of KSHV ORF K9 mutant. **(A).** The primers designed to test the mutation span the KSHV ORF-K9. K9 CDS in RGB-BAC16 is 1,998 bp; the size is reduced to 1,683 bp in K9 mutant contained PSM while that of K9 mutant without PSM is 648 bp.
**(B).** Gel electrophoresis analysis of PCR product amplified with primers listed in S2 Table.
**(C).** The RGB-BAC16 and RGB-K9 Mutant bacmids were digested by *Kpn* I, and then analyzed by gel electrophoresis. The band of RGB-K9-mutant presented a shift of about 1.3 kb.
**(D).** qPCR showing vIRF1, vIRF4 and ORF 57 mRNA expressed in iSLK-RGB-BAC16 and iSLK-RGB-K9 mutant cells.
**(E).** DNA was extracted from HUVECs infected with wild-type virus and mutant virus, amplified with primers listed in S2 Table by PCR, and then analyzed by gel electrophoresis.
**(F).** qPCR showing vIRF4 and ORF 57 mRNA expressed in HUVECs infected with wild-type KSHV (**KSHV_WT**) or vIRF1 mutant virus (**vIRF1_mut**).
**(G).** Western-blotting of phosphorylated p53, acetylated p53, and p21 in HUVECs infected with wild-type KSHV (**KSHV_WT**) or vIRF1 mutant virus (**vIRF1_mut**). The quantified results represent the mean ± SD. *** *P* < 0.001, Student's t-test. undet., undetermined.
(TIF)
