# Correction: Oncogenic KSHV-encoded interferon regulatory factor upregulates HMGB2 and CMPK1 expression to promote cell invasion by disrupting a complex lncRNA-OIP5-AS1/miR-218-5p network

**DOI:** 10.1371/journal.ppat.1009039

**Published:** 2020-10-21

**Authors:** Wan Li, Qingxia Wang, Qi Feng, Fei Wang, Yao Lu, Qin Yan, Shou-Jiang Gao, Chun Lu

Mr. Yao Lu should be included in the author byline. He should be listed as the fifth author, and his affiliation is 1: Department of Microbiology, Nanjing Medical University, Nanjing, P. R. China. The contributions of this author are as follows: Investigation.

The correct citation is: Li W, Wang Q, Feng Q, Wang F, Lu Y, Yan Q, et al. (2019) Oncogenic KSHV-encoded interferon regulatory factor upregulates HMGB2 and CMPK1 expression to promote cell invasion by disrupting a complex lncRNA-OIP5-AS1/miR-218-5p network. PLoS Pathog 15(1): e1007578. https://doi.org/10.1371/journal.ppat.1007578
