# Supplementary material for: Oncogenic KSHV-encoded interferon regulatory factor upregulates HMGB2 and CMPK1 expression to promote cell invasion by disrupting a complex lncRNA-OIP5-AS1/miR-218-5p network
Source: PLoS Pathog. 2019 Jan 30;15(1):e1007578. doi: 10.1371/journal.ppat.1007578 (PMC6370251; doi:10.1371/journal.ppat.1007578)
Supplement: S1 Table — (DOCX) [file ppat.1007578.s001.docx]

**Supplemental Table 1.** Sequences of the siRNAs

| **Gene** | **siRNA No.** | **Sequence of siRNA (5’ to 3’)** |
| --- | --- | --- |
| HMGB2 | si1 | CUGAACAUCGCCCAAAGAUTT |
|  | si2 | CCGUCAAUUUCGCGGAAUUTT |
|  | si3 | GGAGAAGUCGAAGUUUGAATT |
| CMPK1 | si1 | CCGCAUCGUCGAGAAAUAUTT |
|  | si2 | CCUACCUUCAGUCAACAAATT |
|  | si3 | GAGUAGUGGUAGGAGUGAUTT |
| DNMT1 | si1 | GGGACUGUGUCUCUGUUAUTT |
|  | si2 | GCACCUCAUUUGCCGAAUATT |
|  | si3 | CCUCCUGGUUAUGUAGUAATT |
| Dicer | si1 | GCUCGAAAUCUUACGCAAATT |
|  | si2 | CCACACAUCUUCAAGACUUTT |
|  | si3 | CCUCCUGGUUAUGUAGUAATT |
