# Supplementary material for: Oncogenic KSHV-encoded interferon regulatory factor upregulates HMGB2 and CMPK1 expression to promote cell invasion by disrupting a complex lncRNA-OIP5-AS1/miR-218-5p network
Source: PLoS Pathog. 2019 Jan 30;15(1):e1007578. doi: 10.1371/journal.ppat.1007578 (PMC6370251; doi:10.1371/journal.ppat.1007578)
Supplement: S2 Table — (DOCX) [file ppat.1007578.s002.docx]

**Supplemental Table 2.** Primers for deletion and test of ORF-K9 mutagenesis (F, Forward; R, Reverse).

| **Application** | **Primer** |
| --- | --- |
| Deletion | F: 5′-CACTGGACATTGCGGCGCGAGCTAGTCTGGTTGCGGGACA  ATAATACAAAATGCCTACACAGGATGACGACGATAAGTAGGG -3′  R: 5′-CTCCCTCCCATAACAATACGGTGTAGGCATTTTGTATTATTG  TCCCGCAACCAGACTAGCCAACCAATTAACCAATTCTGATTAG -3′ |
| Test | F: 5′- CCCAACATCCATTATGGAAAAACCC -3′  R: 5′- GAAAAGGAAGCTATGTGGTTTCTGG -3′ |
