# Supplementary material for: Oncogenic KSHV-encoded interferon regulatory factor upregulates HMGB2 and CMPK1 expression to promote cell invasion by disrupting a complex lncRNA-OIP5-AS1/miR-218-5p network
Source: PLoS Pathog. 2019 Jan 30;15(1):e1007578. doi: 10.1371/journal.ppat.1007578 (PMC6370251; doi:10.1371/journal.ppat.1007578)
Supplement: S3 Table — (DOCX) [file ppat.1007578.s003.docx]

**Supplemental Table 3.** The sequences of specific primers for qPCR (F, Forward; R, Reverse).

| **Target** | **Application** | **Primer** |
| --- | --- | --- |
| HMGB2 | RT-qPCR | F: 5′- CGGGGCAAAATGTCCTCGTA -3′  R: 5′- CGGAAGAGTCCGGGTGTTT -3′ |
| CMPK1 | RT-qPCR | F: 5′- GGAAGGCAGATGTATCTTTCGTT -3′  R: 5′- TGTTGACTGAAGGTAGGTCTGA -3′ |
| SLIT2 | RT-qPCR | F: 5′- GCGAAGCTATACAGGCTTGAT -3′  R: 5′- TGCAGTCGAAAAGTCCTAAGTTT -3′ |
| SLIT3 | RT-qPCR | F: 5′- GTCAGCGTCATCGAGAGAG -3′  R: 5′- TTCGGCGTGCTCTGGAAAAG -3′ |
| DNMT1 | RT-qPCR | F: 5′- AGAACGGTGCTCATGCTTACA -3′  R: 5′- CTCTACGGGCTTCACTTCTTG -3′ |
| Lnc-OIP5-AS1-1 | RT-qPCR | F: 5′- AGCCACTACCATGATAAGCA -3′  R: 5′- TTTTGTTTAAAATTGGGCCTT -3′ |
| Lnc-OIP5-AS1-2 | RT-qPCR | F: 5′- TCAAGACAGAATGGAGCCTA -3′  R: 5′- AAAAGATGCCAGAAGTACACA -3′ |
| Lnc-OIP5-AS1-3 | RT-qPCR | F: 5′- ATGGCATTCATGATGTCTGA -3′  R: 5′- GTAAAACCTTGAATGTGCTT -3′ |
| Dicer | RT-qPCR | F: 5′- GAGCTGTCCTATCAGATCAGGG -3′  R: 5′- ACTTGTTGAGCAACCTGGTTT -3′ |
| vIRF1  vIRF4  ORF 57 | RT-qPCR  RT-qPCR  RT-qPCR | F: 5′- CCGGACACGACAACTAAGAA -3′  R: 5′- GTCTCTGCGCCATTCAAAAC -3′  F: 5′- CGTTAGCCACTCCGGTCCC -3′  R: 5′- TCCACGAAGGTTCCCATACACA -3′  F: 5′- GACAGGGATATCACCGCTCT -3′  R: 5′- GCAACTCACCCAGTACGCTTG -3′ |
