# Supplementary figures and images for: Oncogenic KSHV-encoded interferon regulatory factor upregulates HMGB2 and CMPK1 expression to promote cell invasion by disrupting a complex lncRNA-OIP5-AS1/miR-218-5p network
Source: PLoS Pathog. 2019 Jan 30;15(1):e1007578. doi: 10.1371/journal.ppat.1007578 (PMC6370251; doi:10.1371/journal.ppat.1007578)

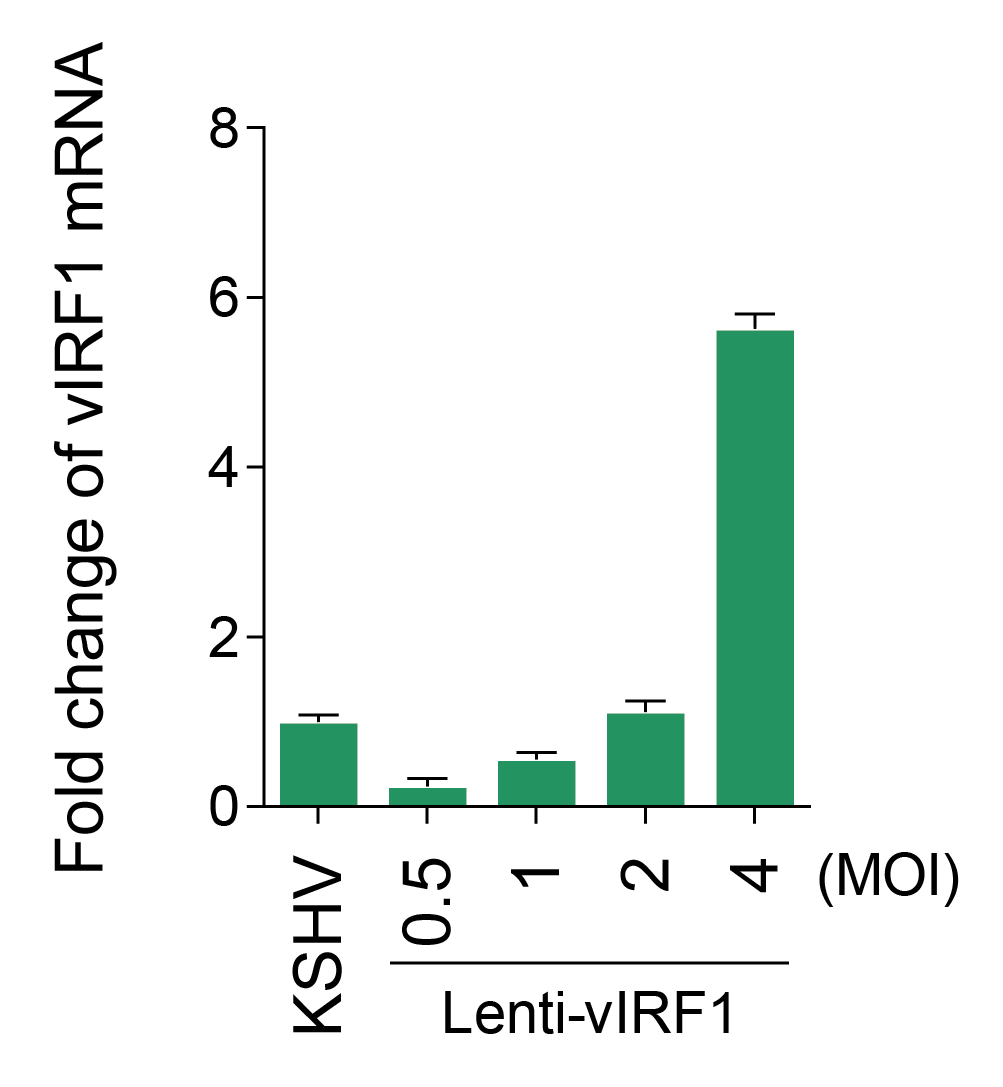

Supplement: S1 Fig — qPCR results showing vIRF1 mRNA expression in HUVECs infected with KSHV or transduced with different MOI of lentiviral vIRF1. The level of vIRF1 mRNA in KSHV cells was set as ‘‘1” for comparison. The quantified results represent the mean ± SD. (TIF) [file ppat.1007578.s004.tif]

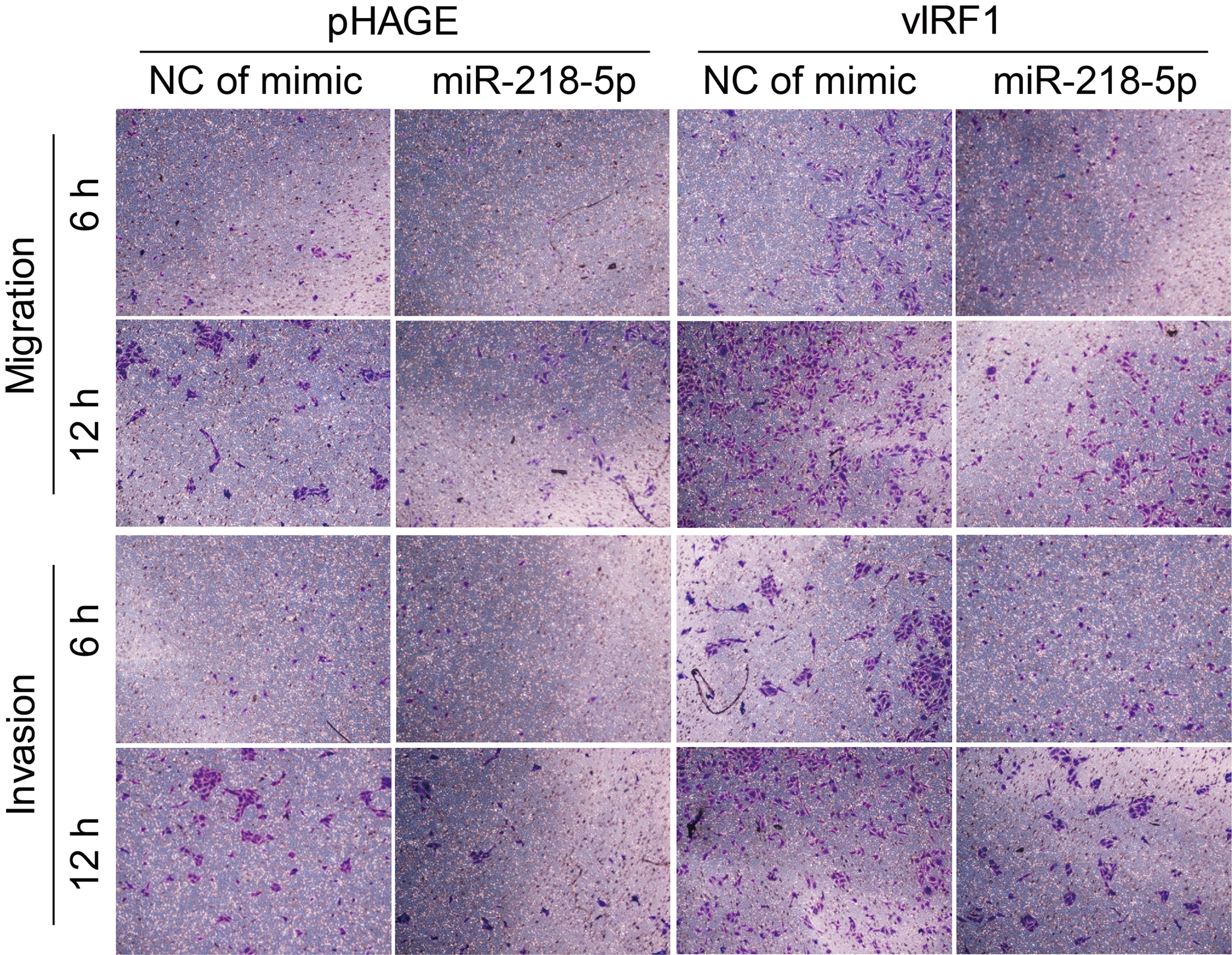

Supplement: S2 Fig — Representative images of migration and invasion analysis of vIRF1-infected HUVECs transfected with mimics of miR-218-5p for 48 h. Original magnification, ×100. (TIF) [file ppat.1007578.s005.tif]

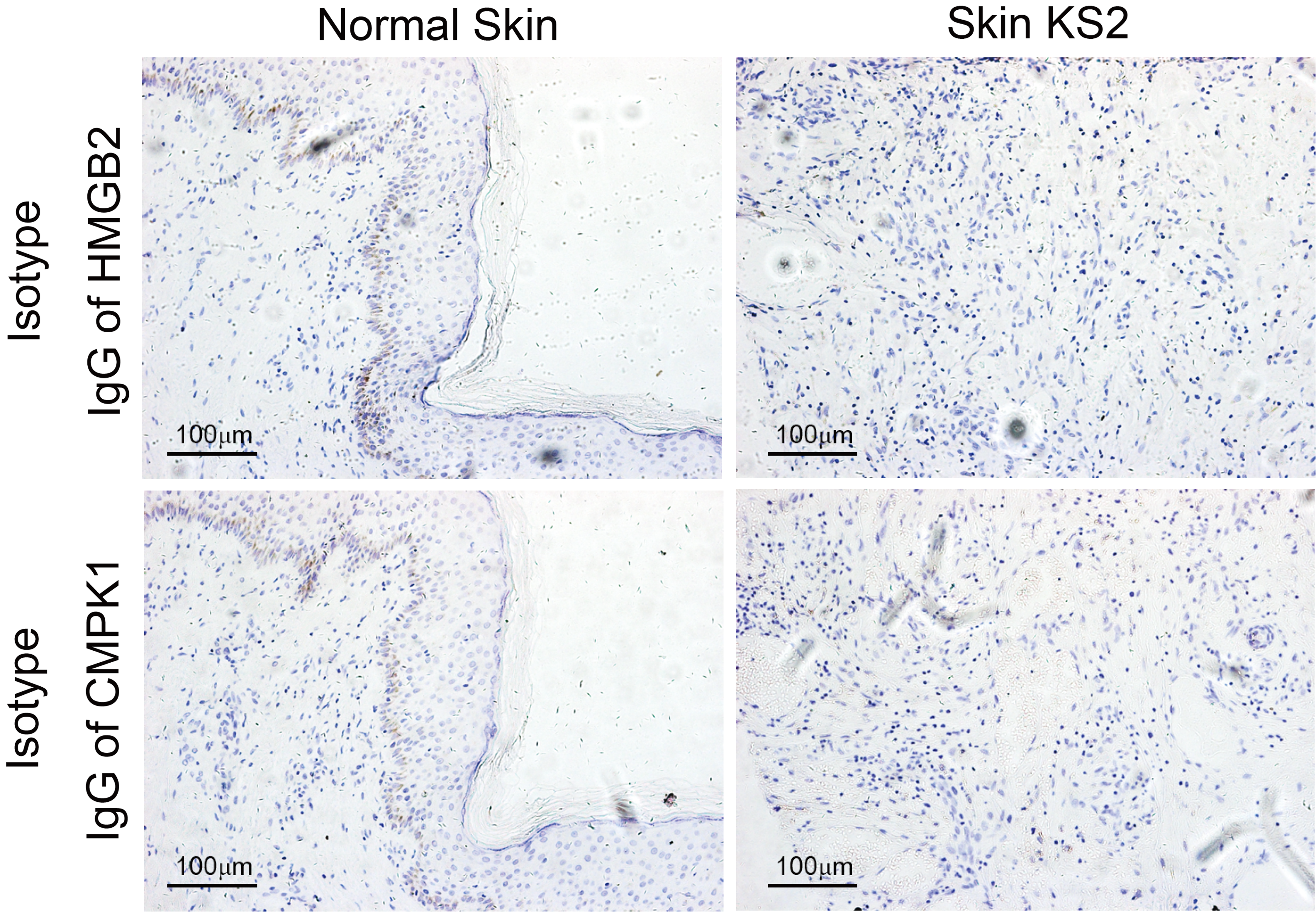

Supplement: S3 Fig — Immunohistochemical staining of isotype control immunoglobulin G (IgG) for HMGB2 (Isotype IgG of HMGB2) and CMPK1 (Isotype IgG of CMPK1) in normal skin, and skin KS of patient #2 (Skin KS2). Magnification, ×200, ×400. (TIF) [file ppat.1007578.s006.tif]

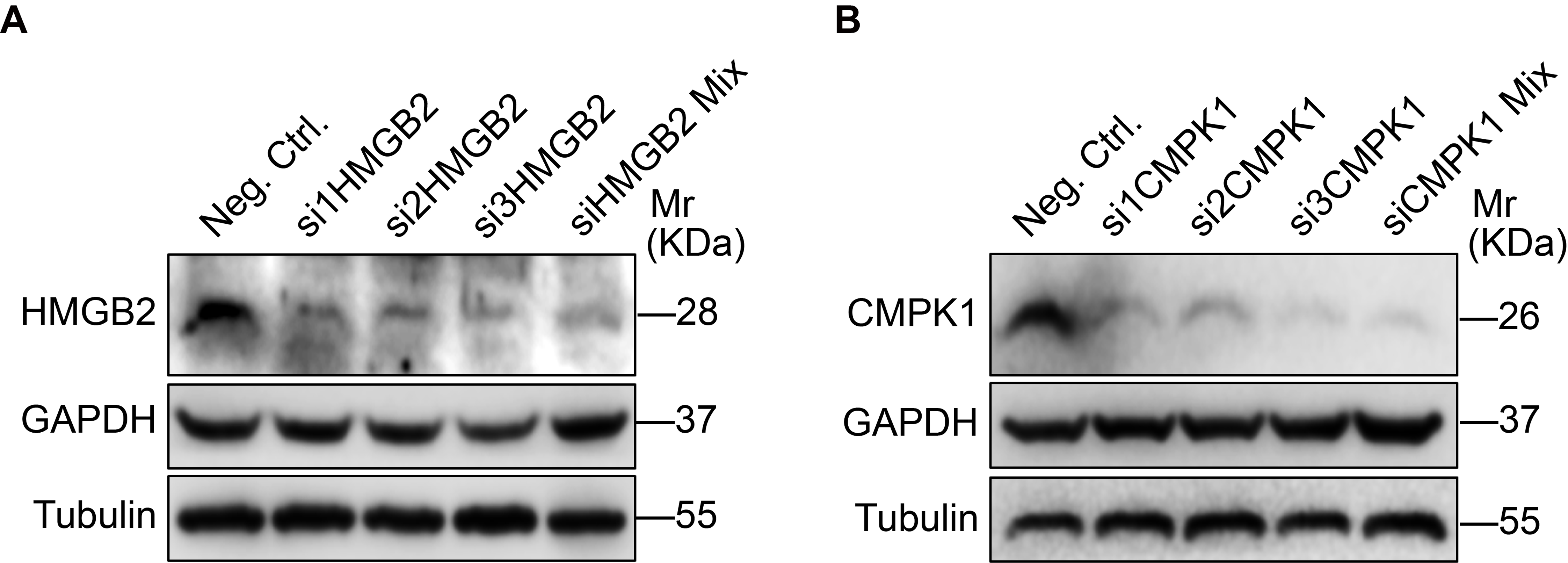

Supplement: S4 Fig — (A). Western-blotting of HMGB2 in HUVECs transfected with No.1 (si1HMGB2), No. 2 (si2HMGB2), No. 3 (si3HMGB2), and a mixture of No. 1, 2 and 3 (siHMGB2 Mix) siRNAs targeting HMGB2. (B). Western-blotting of CMPK1 in HUVECs transfected with No.1 (si1CMPK1), No. 2 (siCMPK1), No. 3 (si3CMPK1), and a mixture of No. 1, 2 and 3 (siCMPK1 Mix) siRNAs targeting CMPK1. (TIF) [file ppat.1007578.s007.tif]

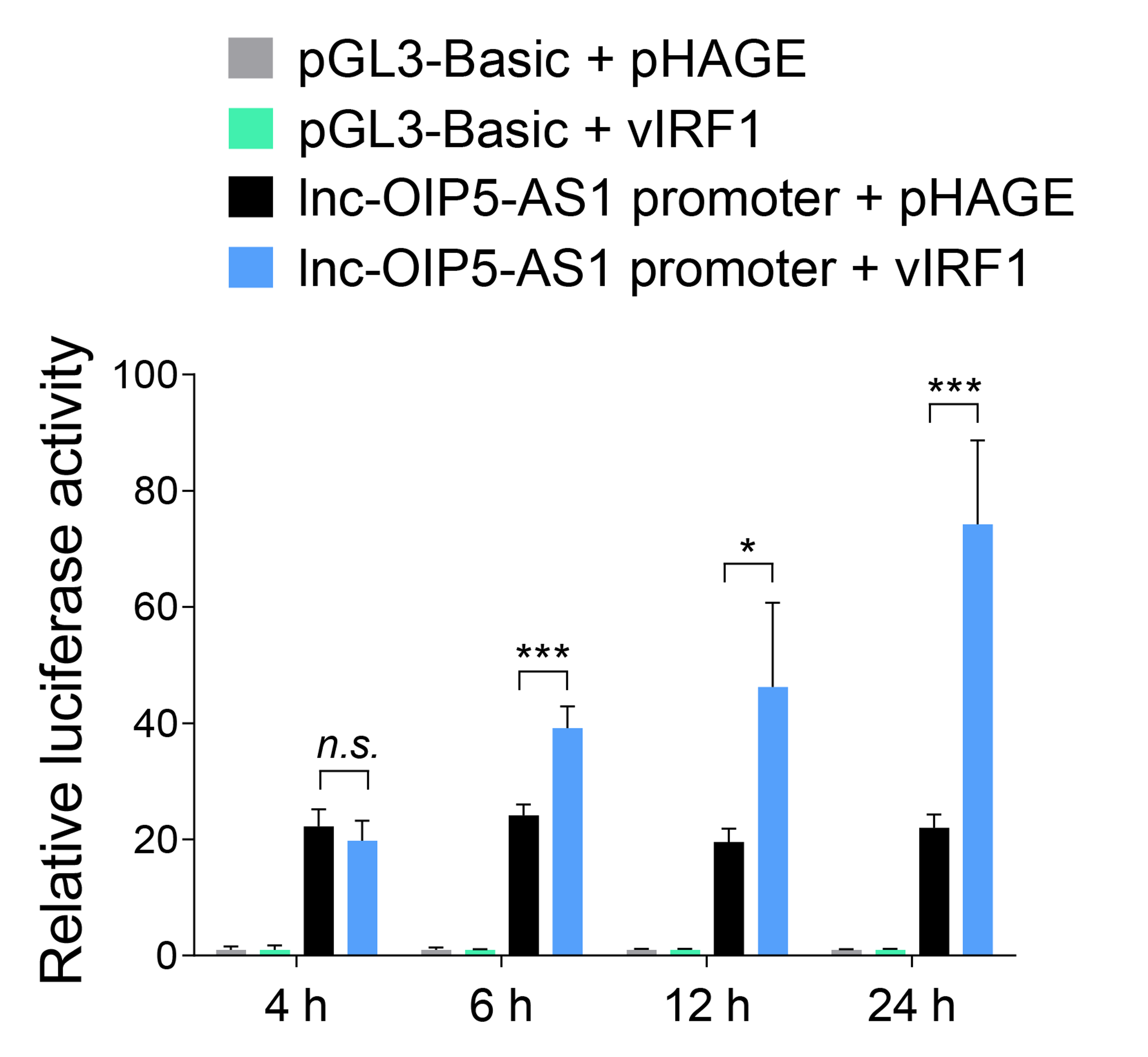

Supplement: S5 Fig — Luciferase activity in HEK293T cells cotransfected with vIRF1 and the lnc-OIP5-AS1 promoter reporter for 4 h, 6 h, 12 h and 24 h, respectively. The quantified results represent mean ± SD. * P < 0.05, *** P < 0.001, Student's t-test. n.s, not significant. (TIF) [file ppat.1007578.s008.tif]

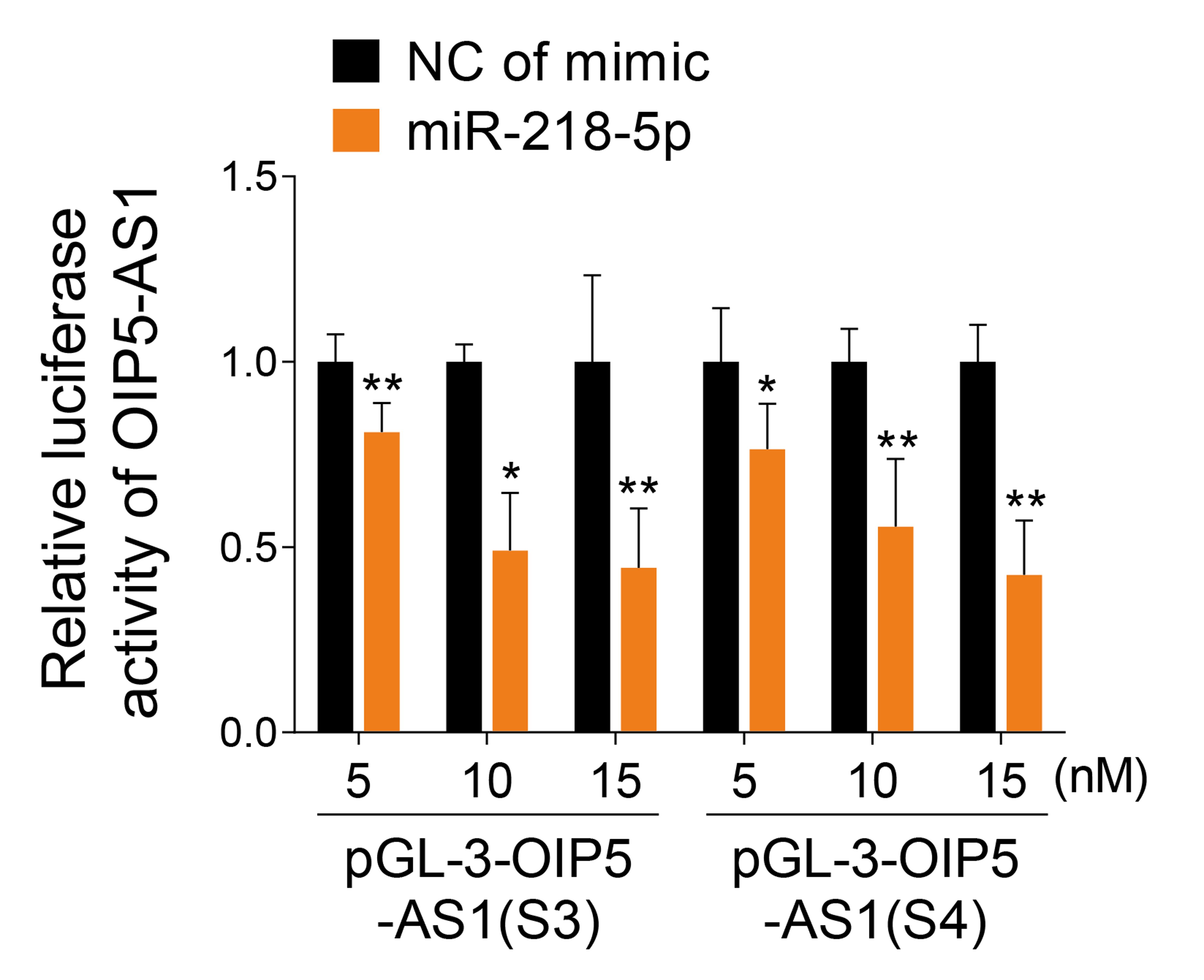

Supplement: S6 Fig — Luciferase activity in HEK293T cells cotransfected with an incremental amount of miR-218-5p mimic (miR-218-5p) (5, 10, and 15 nM) or its control (NC of mimic) together with pGL-3-OIP5-AS1(S3) or pGL-3-OIP5-AS1(S4) reporter for 48 h. The quantified results represent mean ± SD. * P < 0.05, ** P < 0.01, Student's t-test. (TIF) [file ppat.1007578.s009.tif]

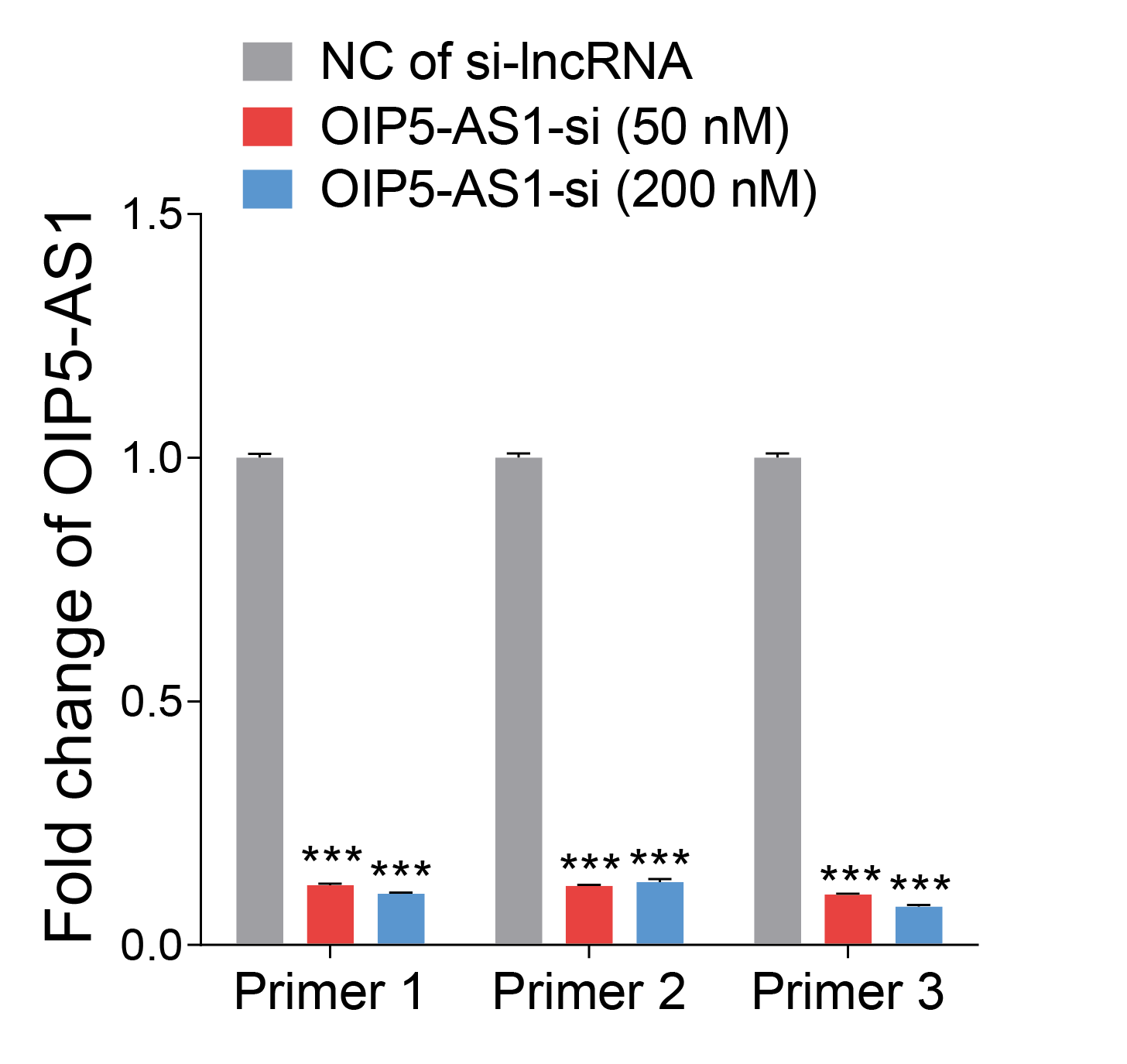

Supplement: S7 Fig — qPCR showing lnc-OIP5-AS1 expression in HUVECs transfected with an incremental amount of lncRNA Smart Silencer targeting lnc-OIP5-AS1 (OIP5-AS1-si) (50 and 200 nM) for 48 h. Three specific primers of lnc-OIP5-AS1 were used. The quantified results represent mean ± SD. *** P < 0.001, Student's t-test. (TIF) [file ppat.1007578.s010.tif]

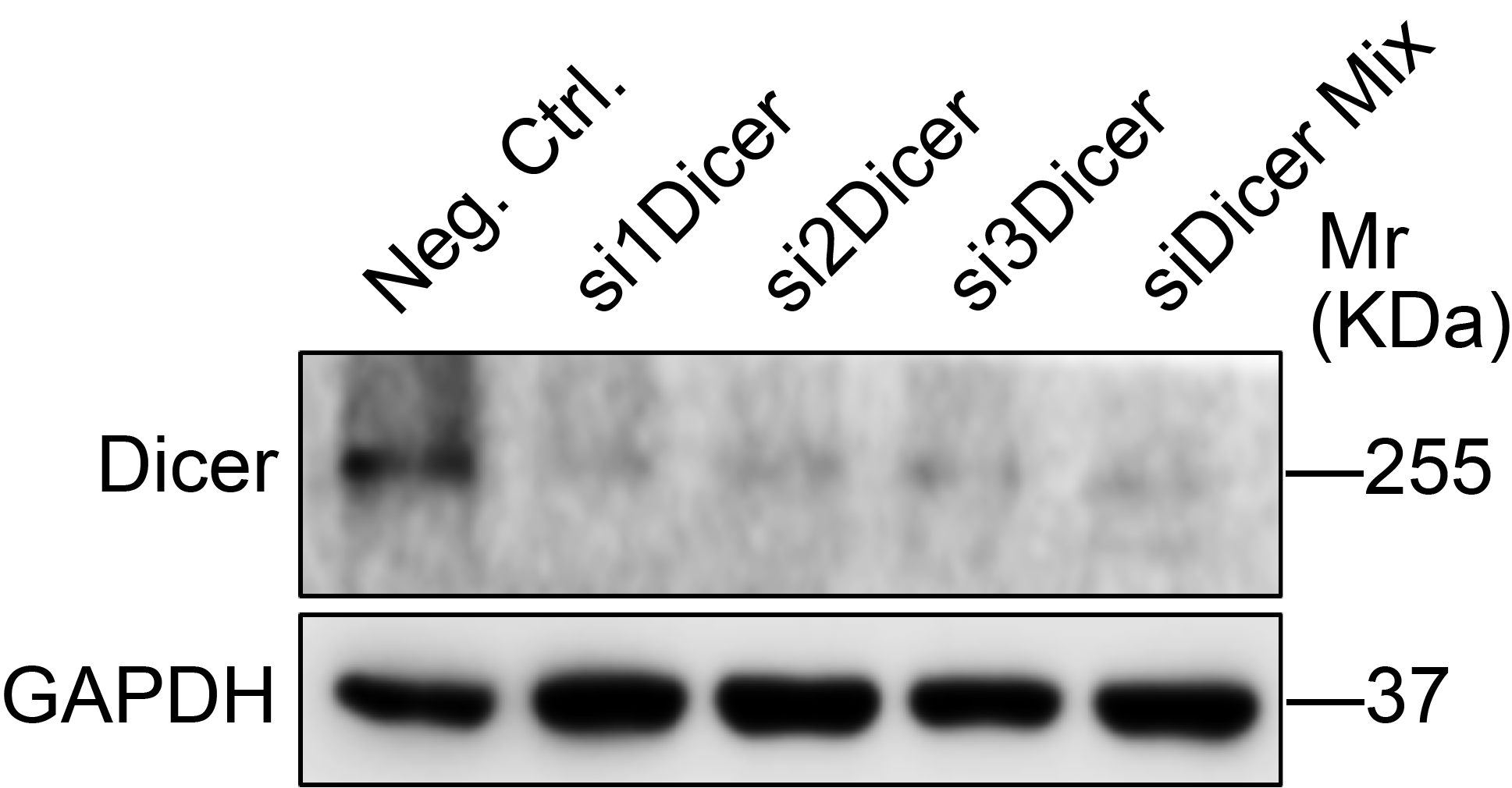

Supplement: S8 Fig — Western-blotting of Dicer in HUVECs transfected with No.1 (si1Dicer), No. 2 (si2Dicer), No. 3 (si3Dicer), and a mixture of No. 1, 2 and 3 (siDicer Mix) siRNAs targeting Dicer. (TIF) [file ppat.1007578.s011.tif]

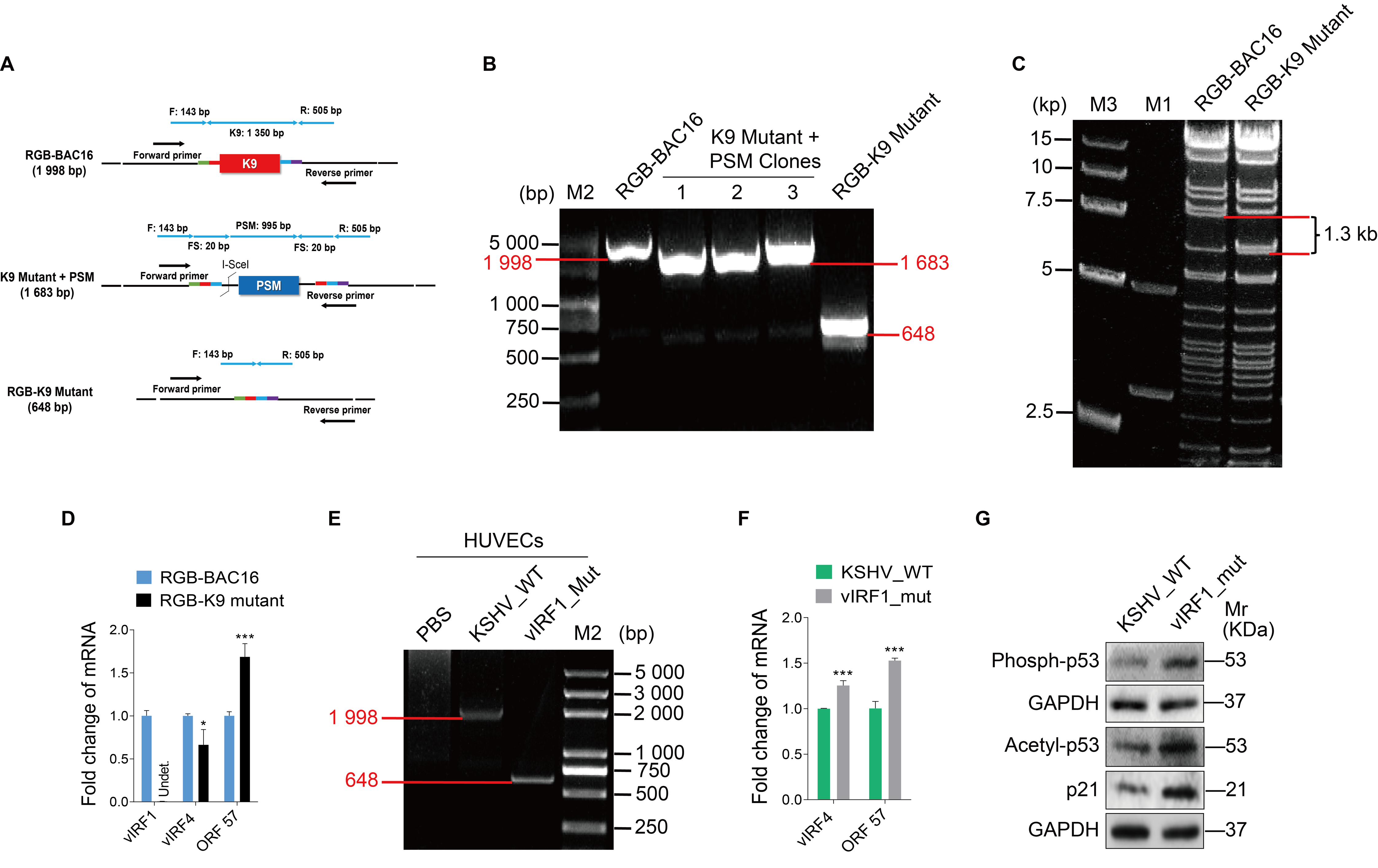

Supplement: S9 Fig — (A). The primers designed to test the mutation span the KSHV ORF-K9. K9 CDS in RGB-BAC16 is 1,998 bp; the size is reduced to 1,683 bp in K9 mutant contained PSM while that of K9 mutant without PSM is 648 bp. (B). Gel electrophoresis analysis of PCR product amplified with primers listed in S2 Table. (C). The RGB-BAC16 and RGB-K9 Mutant bacmids were digested by Kpn I, and then analyzed by gel electrophoresis. The band of RGB-K9-mutant presented a shift of about 1.3 kb. (D). qPCR showing vIRF1, vIRF4 and ORF 57 mRNA expressed in iSLK-RGB-BAC16 and iSLK-RGB-K9 mutant cells. (E). DNA was extracted from HUVECs infected with wild-type virus and mutant virus, amplified with primers listed in S2 Table by PCR, and then analyzed by gel electrophoresis. (F). qPCR showing vIRF4 and ORF 57 mRNA expressed in HUVECs infected with wild-type KSHV (KSHV_WT) or vIRF1 mutant virus (vIRF1_mut). (G). Western-blotting of phosphorylated p53, acetylated p53, and p21 in HUVECs infected with wild-type KSHV (KSHV_WT) or vIRF1 mutant virus (vIRF1_mut). The quantified results represent the mean ± SD. *** P < 0.001, Student's t-test. undet., undetermined. (TIF) [file ppat.1007578.s012.tif]
